# Enhancing Microwave Dynamic Effects via Surface States of Ultrasmall 2D MOF Triggered by Interface Confinement for Antibiotics‐Free Therapy

**DOI:** 10.1002/advs.202300084

**Published:** 2023-05-18

**Authors:** Yuqian Qiao, Shuilin Wu, Yufeng Zheng, Chaofeng Wang, Zhaoyang Li, Yu Zhang, Shengli Zhu, Hui Jiang, Zhenduo Cui, Xiangmei Liu

**Affiliations:** ^1^ School of Materials Science and Engineering Peking University Beijing 100871 P. R. China; ^2^ School of Health Science and Biomedical Engineering Hebei University of Technology Xiping Avenue 5340, Beichen District Tianjin 300401 P. R. China; ^3^ Biomedical Materials Engineering Research Center Hubei Key Laboratory of Polymer Materials Ministry‐of‐Education Key Laboratory for the Green Preparation and Application of Functional Materials School of Materials Science and Engineering Hubei University Wuhan 430062 P. R. China; ^4^ School of Materials Science and Engineering The Key Laboratory of Advanced Ceramics and Machining Technology by the Ministry of Education of China Tianjin University Tianjin 300072 P. R. China; ^5^ Department of Orthopedics Guangdong Provincial People's Hospital Guangdong Academy of Medical Sciences Guangzhou 510080 P. R. China

**Keywords:** 2D metal—organic framework, antibiotic‐free, microwave dynamic therapy, microwaveocaloric therapy, surface states

## Abstract

Microwave (MV)‐trigged dynamic therapy based on MV‐responsive materials is promising for treating deep infection diseases that cannot be effectively treated by antibiotics, like life‐threatening osteomyelitis. Surface states of materials affect the generation of free charges under the excitation source with energy less than the band gap, consequently influencing the MV dynamic effects. Herein, an MV responsive system with interface confined 2D metal–organic framework (2D MOF) on oxidized carbon nanotube (CNT) is prepared, in which the ultrasmall Cu‐based 2D MOF possesses sufficient surface/interface defects, endowing the system a large number of surface states. Under MV irradiation, the synthesized CNT‐2D MOF not only efficiently absorbs and converts the microwave into heat for microwaveocaloric therapy (MCT) via enhanced hetero‐interfacial polarization, but also generates excited electrons via surface state for microwave dynamic therapy (MDT). This biocompatible CNT‐2D MOF exhibits highly effective broad‐spectrum antimicrobial activity against seven pathogenic bacteria, including Gram‐negative and Gram‐positive pathogens, under 7 min MV irradiation. And this system is proven to efficiently eradicate *Staphylococcus aureus* infected rabbit tibia osteomyelitis. Significantly, MV‐excited MCT and MDT of CNT‐CuHHTP developed in this study makes a major step forward in antibiotic‐free MV therapy in deep tissue bacterial infection diseases.

## Graphical abstract

Antibiotic‐free deep antibacterial strategy. CNT‐CuHHTP designed through interface confinement engineering exhibits broad‐spectrum antibacterial properties under microwave irradiation and can eliminate *Staphylococcus aureus*‐infected osteomyelitis by synergistic microwave thermotherapy and microwave kinetic therapy.

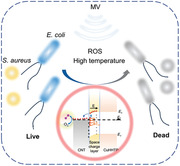

## 1 Introduction

Deep tissue infection diseases, for example, osteomyelitis, induced by pathogenic bacteria has threatened human health.^[^ ^1^ ^]^ Without conducting a proper treatment in time, it may lead to life‐threatening sepsis with severe organ failure.^[^ ^2^ ^]^ Clinical treatment always requires multiple invasive surgeries and long‐term antibiotic therapy, which brings not only pains to patients, but also serious side effects and even drug resistance caused by antibiotics.^[^ ^3^ ^]^ In addition, an antibiotic‐free therapy, phototherapy, including photothermal therapy and photodynamic therapy, is ineffective for the treatment of deep tissue infections limited by the poor penetration of light.^[^ ^4^ ^]^ Hence, it is urgent to develop an non‐invasive antibiotic‐free strategy for deep tissue infections.

Microwave (MV), as an electromagnetic wave with a longer wavelength than light, has a strong penetration ability and is a promising exogenous energy source to assist treatment of deep tissue infection diseases. Recently, microwaveocaloric therapy (MCT) with high heating efficiency and deep tissue penetration ability has been widely used in clinical practice.^[^ ^5^ ^]^ Microwave sensitizer makes a main role on absorbing and converting the MV into microwaveocaloric via the dielectric loss or magnetic loss for improved MCT. Oxidized carbon nanotube (CNT) with large aspect ratio is easy to form a conductive network structure, which is conducive to the absorption mechanism of multiple reflections. Moreover, the large specific surface area and small size effect of CNT increase the crystal defects and dangling bonds due to the high proportion of surface atoms, which is conducive to the increase of dielectric loss due to dipole polarization.^[^ ^6^ ^]^ However, the incident MV is severely reflected and cannot be converted into heat for MCT, when CNT is used alone due to the skin effect caused by its high conductivity.^[^ ^7^ ^]^ Coating a layer of material with moderate conductivity on the surface of CNT can effectively adjust the wave resistance matching by heterointerfacial polarization while maintaining the high dielectric loss and network‐like structure, which are conducive to efficient MCT. In addition, unlike most MOFs with poor conductivity, the metal ions in CuHHTP and aromatic organic ligands will form abundant *π*–*π*/*π*–d conjugated structures when they are bonded, presenting delocalized electrons similar to graphene, thus producing high conductivity.^[^ ^8^ ^]^ Moreover, CuHHTP usually contains a lot of micropores and has a large specific surface area, which increases the active sites of the reaction to a certain extent. As a 2D layered conductive metal–organic framework, the conductivity of CuHHTP is much weaker than CNT,^[^ ^9^ ^]^ making this 2D MOF avoid the skin effect. In addition, CuHHTP is composed of copper atoms and 2,3,6,7,10,11‐hexahydroxytriphenylene (HHTP) ligands, its intrinsically porous structure is conducive to the absorption of MV. Furthermore, the organic–inorganic hybrid structure commonly leads to a large number of unsaturated sites (e.g., unsaturated Cu—O_2_ centers) and many dangling bonds (e.g., —OH), which are beneficial to the dipole polarization consumption of MV for improved MCT.^[^ ^10^ ^]^

On the contrary, the development of microwave dynamic therapy (MDT) has been limited because the MV energy is only 10^−3^ eV, which is not enough to activate bacterial‐toxic reactive oxygen species (ROS) generation similar to photodynamic therapy in theory.^[^ ^11^ ^]^ In practice, there are a wide variety of surface states existing in the forbidden band of the semiconductor, due to the surface effect including surface steps, unsaturated bonds, and adsorbed atoms/molecules on the surface of the material.^[^ ^12^ ^]^ These surface states correspond to different surface energy levels, with multi‐level characteristics distributed in the forbidden band, and the energy levels matching the microwave energy may appear in the multi‐level status.

Therefore, we speculate that these surface energy levels could alleviate the hopping difficulty of electrons through multi‐level transitions, which will promote the generation of excited electrons under microwave stimulation and achieve an enhanced MDT. The above mentioned CuHHTP commonly possesses unsaturated bonds (e.g., Cu—O_2_ centers), which is the key to generate surface states on its surface. Meanwhile, the oxygen‐containing functional groups on CNT provide nucleation sites that allow 2D MOF to nucleate and then grow into MOF crystals. Because of the steric hindrance effect, the epitaxial growth of CuHHTP will be affected. Therefore, when CNT is combined with CuHHTP, CNT will limit the infinite epitaxial growth of CuHHTP, so that the CuHHTP sheet smaller and has more surface states, which is conducive to efficient microwave dynamic therapy.

Herein, we designed a CNT‐CuHHTP heterojunction with rich surface states caused by confinement effect, which showed a synergistic effect of microwaveocaloric and microwave dynamic therapy. CNT‐CuHHTP has excellent microwaveocaloric effect due to its excellent electrical conductivity, multiple reflections, heterointerfacial polarization, and dipole polarization. Importantly, under MV excitation, the CNT‐CuHHTP can generate free electrons through surface energy level transitions, while the built‐in electric field formed in the heterojunction accelerates the electron transfer, further enabling efficient MV dynamic property and generating a large amount of ·O_2_^−^. Therefore, CNT‐CuHHTP can rapidly and efficiently eradicate osteomyelitis by MCT and MDT. The surface states engineering‐guided generation of microwave‐excited electrons may bring a novel insight for developing microwave energy absorption and converting materials for various kinds of applications such as microwaveocaloric and microwave dynamic therapy of deep tissue infectious diseases.

## 2 Results and Discussion

### 2.1 Synthetic Procedure and Microscopic Structural of CNT‐CuHHTP

**Figure 1:**
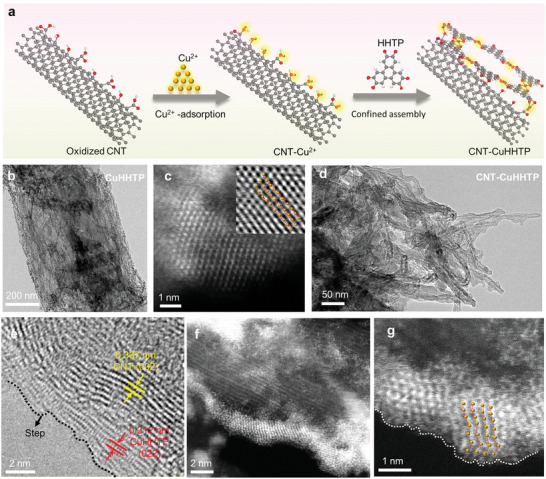
Synthetic procedure and microscopic structural of CNT‐CuHHTP. a) Schematic illustration for the synthesis of CNT‐CuHHTP. b) Typical TEM image of CuHHTP. c) HAADF‐STEM image of CuHHTP. Inset: partial enlarged view of (c). d) Typical TEM image of CNT‐CuHHTP. e) Typical HRTEM image of CNT‐CuHHTP. f) HAADF‐STEM image of CNT‐CuHHTP. g) Partial enlarged view of (f). The yellow ball represents the copper atom.

The “electric‐wire like” CNT‐CuHHTP was prepared via hydrothermal method. In this reaction, the C/O functional groups (—COOH, —C—OH) of CNT could absorb the free Cu^2+^ via electrostatic attraction, thus forming nucleation centers to guide the crystal and growth of Cu‐MOF. After HHTP ligands coordinated to Cu^2+^, the bridge chain has self‐assembled into 2D Cu‐MOF along with CNT skeleton (**Figure** **1**a). Instead, the free nucleation and growth of CuHHTP is formed by layer‐by‐layer stacking structures comprised of aggregated 2D nanosheets with a lateral size of 0.2–1 µm, as observed in Figure 1b and Figure S1, Supporting Information. High‐angle annular dark‐field scanning transmission electron microscopy (HAADF‐STEM) image of CuHHTP showed that Cu atoms are arranged in order (Figure 1c) and the lattice spacing is uniform (Figure 1c, inset). When CNT (Figure S2, Supporting Information) was introduced to reaction system, the ultrasmall CuHHTP is formed and grow along the CNT skeleton (Figure 1d), revealing the CNT can confine the epitaxial growth of CuHHTP. The dark‐field TEM image and the corresponding elemental mapping images of CNT‐CuHHTP in Figure S3, Supporting Information, displayed a similar distribution area of individual elements of C, O, and Cu, preliminarily confirming that the CuHHTP covered the surface of CNT. The high‐resolution transmission electron microscopy (HRTEM) image of the CNT‐CuHHTP in Figure 1e exhibited the lattice distance of 0.312 nm and 0.337 nm, which belonged to the (022) and (002) lattice plane of CuHHTP and CNT, respectively. Notably, obvious steps and defects appears in the CNT‐CuHHTP. Further, the HAADF‐STEM image displayed the high‐density of Cu single atoms can be clearly identified along the CNT skeleton, noticeably, ultrasmall CuHHTP shows discontinuous and irregular epitaxial growth (Figure 1f) and lattice distortion (Figure 1g). Additionally, the lattice mismatch at the heterojunction leads to the appearance of interface states. These surface/interface effects enhanced by CNT confinement are the main source of CNT‐CuHHTP surface states.

### 2.2 Structural Characterization of CNT‐CuHHTP

**Figure 2:**
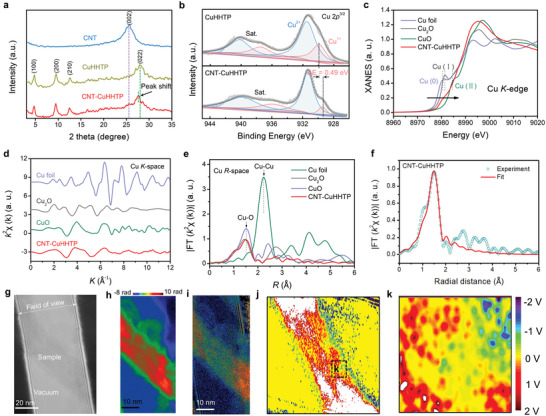
The structural characterizations of CNT‐CuHHTP. a) The XRD patterns of CNT, CuHHTP, and CNT‐CuHHTP. b) The Cu 2p XPS spectra in CuHHTP and CNT‐CuHHTP. c) Synchrotron radiation Cu K‐edge XANES. d) The k^2^ *χ*(k)‐oscillation curves and e) the Fourier transform curves of Cu K‐edge EXAFS spectra for Cu foil, Cu_2_O, CuO, and CNT‐CuHHTP. f) FT‐EXAFS fitting curve of CNT‐CuHHTP in R space. g) Electron holograms of CNT‐CuHHTP. h) Reconstructed phase image of (g). i) Relative thickness image of CNT‐CuHHTP was converted by amplitude. j) Reconstructed potential map. k) Enlarged view of the black boxed region in (j).

The X‐ray diffraction (XRD) pattern of the CNT‐CuHHTP (**Figure** **2**a) exhibits almost the same peaks as those of the pure CuHHTP except for the peak at 2*θ* = 26°, which is assigned to the (002) plane of the graphite‐like structure of CNT, indicating the coexistence of CuHHTP and CNT in the CNT‐CuHHTP. Especially, the position of CuHHTP (022) peak after CNT doping moves from 28.0° to 27.7°, and the corresponding lattice spacing increases from 0.309 to 0.312 nm. This is mainly due to the lattice distortion of ultrasmall CuHHTP caused by the confinement effect. Besides, the X‐ray photoelectron spectroscopy (XPS) full spectrum (Figure S4, Supporting Information) of CNT‐CuHHTP demonstrated the existence of C, O, and Cu in CNT‐CuHHTP, which is consistent with the element mapping results. The high‐resolution spectrum of Cu 2p^3/2^ spectrum was deconvoluted into Cu^2+^ and Cu^1+^, and the two corresponding satellite peaks were shown in Figure 2b. A clear negative shift of Cu^1+^ (≈0.49 eV) binding energy was observed in CNT‐CuHHTP compared to the one in CuHHTP, further suggesting a strong chemical interaction between CuHHTP and CNT. These results illustrate the successful construction of CNT‐CuHHTP heterostructure.

To explore the intrinsic atomic structure of CNT‐CuHHTP, synchrotron radiation‐based X‐ray absorption fine structure (XAFS) was performed. The Cu K‐edge shows the absorption edge positions of copper in CNT‐CuHHTP is between Cu foil and CuO references, means that the average valance state of copper in CNT‐CuHHTP is between metallic Cu^0^ and oxidized Cu^2+^ (Figure 2c), which is consistent with the XPS results. CNT‐CuHHTP maintains higher energy beyond Cu_2_O, suggesting more positive charges carried by copper atoms of the CNT‐CuHHTP. The *k* ^2^ *χ*(*k*) oscillation curve of CNT‐CuHHTP at Cu K‐edge shows a different trend in shape and oscillating frequency to that of control samples over the whole range of 0–12 Å^−1^ (Figure 2d; Figure S5, Supporting Information), inferring different structural configurations of Cu in both CNT‐CuHHTP and control samples. One dominant peak at about 1.50 Å in the Fourier transformed curves of Cu K‐edge extended XAFS (FT‐EXAFS) (Figure 2e) is contributed from the nearest coordination shell of Cu‐O bonds, is observed for CNT‐CuHHTP. Notably, CNT‐CuHHTP shows a weaker Cu—O peak relative to that of CuO, possibly indicating the presence of partial coordinatively unsaturated Cu nodes. Moreover, the average oxidation state of Cu species in CNT‐CuHHTP is confirmed as 1.7 based on the X‐ray absorption near‐edge spectra (XANES) analyses (Figure S6, Supporting Information), obviously lower than that of CuO (about 2), which again suggests the existence of unsaturated Cu nodes in CNT‐CuHHTP. From the EXAFS fitting results (Figure 2f; Table S1, Supporting Information), the coordination number of Cu—O bonds is quantitatively determined to be about 3.46 for CNT‐CuHHTP, evidently confirming the coexistence of unsaturated Cu—O_2_ and saturated Cu—O_4_ centers. The wavelet transforms (WT) contour plots of Cu foil displayed the maximum intensity at 7.0 Å^−1^ (Figure S7a, Supporting Information) due to their corresponding Cu—Cu coordination. And the WT contour plots of Cu_2_O and CuO displayed the maximum intensity at 4.2 Å^−1^ (Figure S7b, Supporting Information) and 5.0 Å^−1^ (Figure S7c, Supporting Information), respectively, which could be due to their corresponding Cu—O coordination. Compared to this, the maximum intensity was found to be positioned at 4.8 Å^−1^ in the WT contour plot of CNT‐CuHHTP, which is ascribed to the coordination between C/O (CNT) and Cu (Figure S7d, Supporting Information). Accordingly, on the basis of the XAFS results, CNT‐CuHHTP have abundant unsaturated Cu nodes. This defective structure endow CNT‐CuHHTP with abundant surface energy levels and higher oxygen adsorption capability, which might be beneficial to the microwave dynamic process.

In our work, electron holography measurements with high sensitivity (0.36 nm spatial resolution and 0.1 V voltage resolution, Figure S8, Supporting Information) were aimed to detect the electrical potential of CNT‐CuHHTP. Specifically, according to the following formula:

(1) $$\Delta \theta \;\left(x,y\right)=C_{E\;}\;V_{p}\left(x,y\right)t$$

We can deduce that the projected average electric potential (*V*p) of the sample is determined by its reconstructed phase shift (Δ*θ*) and thickness (*t*), where *C*_E_ is the interaction constant (0.00728 rad V^−1^ nm^−1^ for 200 kV accelerating voltage).^[^ ^13^ ^]^ Following this method, a representative electron hologram of CNT‐CuHHTP (sample) is shown in field of view with a vacuum region (Figure 2g). The reconstructed phase map (Figure 2h) with the desired information about the potential distribution shows the phase shift relative to vacuum. Moreover, the phase shift of CNT and CuHHTP can be more clearly observed from their local magnified images (Figure S9a,b, Supporting Information) and corresponding local linear phase images (Figure S9c,d, Supporting Information). The relative thickness information of the sample obtained from the amplitude is shown in Figure 2i. Therefore, all parameters for obtaining the potential map are available. In this way, an electrostatic potential difference ranging between −2 and 2 V, which can be demonstrably observed across the interfaces inside CNT‐CuHHTP (Figure 2j). And a distinct potential distribution can be noticed near the interface of CNT and CuHHTP, showing a positive potential on the CNT side and negative potential on the CuHHTP side, which facilitates the separation and transfer of free electrons at the heterointerface to enhance the microwave kinetics effect. In addition, the inhomogeneous potential distribution (Figure 2k) favors the dense interfacial polarization at phase boundary for enhanced MCT.

### 2.3 Microwaveocaloric Performance and Mechanism of CNT‐CuHHTP

**Figure 3:**
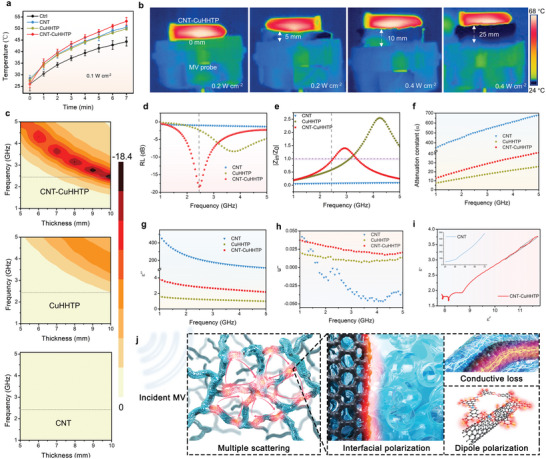
Microwaveocaloric performance and mechanism of CNT‐CuHHTP. a) The microwaveocaloric curves of CNT, CuHHTP, and CNT‐CuHHTP under MV excitation for 7 min. Data are presented as mean ± standard deviations from a representative experiment (*n*  =  3 independent samples). b) Infrared thermal images of CNT‐CuHHTP (1 mg mL^−1^) through different thicknesses (0, 5, 10, and 25 mm) of pork tissue under MV excitation. c) Contour map of RL values of CNT, CuHHTP, and CNT‐CuHHTP. d) RL values of CNT, CuHHTP, and CNT‐CuHHTP at 1–5 GHz (10 mm). e) Frequency dependence of relative input impedance (|*Z*_in_/*Z*_0_|) and f) attenuation constant *α*. g) Imaginary part of complex permittivity and h) permeability of the three samples in the 1–5 GHz range. i) Plots of *ε*′ versus *ε*″ for CNT‐CuHHTP. Inset: plots of *ε*′ versus *ε*″ for CNT. j) Microwave absorption mechanism of CNT‐CuHHTP.

We tested the microwaveocaloric performance of different materials under medical low‐power MV intensity (2.45 GHz, 0.1 W cm^−2^). As shown in **Figure** **3**a, the temperature of CNT‐CuHHTP solution rose from 25.9 °C to as high as 53.1 °C within 7 min, higher than that of the group of physiological saline (Ctrl, 44.3 °C), pure CuHHTP (49.8 °C), and CNT (50.5 °C). The temperature can be controlled by adjusting the MV power. When the MV power is changed from 0.1 to 0.2 W cm^−2^, the saline solution of CNT‐CuHHTP can even reach up to 68 °C after 7 min MV irradiation (Figure 3b, 0 mm). To verify whether the microwaveocaloric effect of CNT‐CuHHTP can be used for in situ hyperthermia after penetration different thicknesses of tissue, pork tissues of 5, 10, and 25 mm thicknesses were selected as the model tissues. As the corresponding infrared thermal images shown in Figure 3b, under MV, the temperature of CNT‐CuHHTP solution can still rise up to 68 °C even though the pork tissue is 25 mm thick by increasing MV power to 0.4 W cm^−2^, which is close to the temperature of CNT‐CuHHTP solution without pork. Importantly, under the same conditions, MV irradiation does not cause hyperthermia in the surrounding tissues of the CNT‐CuHHTP (Figure 3b). That is to say, by adjusting the MV power, the CNT‐CuHHTP solution can achieve desired microwaveocaloric performance even at 25 mm penetration depth without causing hyperthermia in the surrounding tissues, which is the key for the successful treatment of deep tissue infection.

To reveal the mechanism of the efficient microwaveocaloric performance of CNT‐CuHHTP, we tested its MV absorption and energy conversion properties using the reflection loss (RL) value, where a lower RL value means stronger MV absorption and more efficient energy conversion capacity.^[^ ^14^ ^]^ CNT‐CuHHTP has the lower RL value than CNT and CuHHTP under different test conditions (Figure 3c). Specifically, the RL value of CNT‐CuHHTP reached −18.42 dB (≈98.56% absorption) at 2.45 GHz, while the RL value of CNT and CuHHTP is only −0.98 dB (≈20.20% absorption) and −2.29 dB (≈40.98% absorption), respectively (Figure 3d). The above results indicated the excellent MV response properties of the synthesized CNT‐CuHHTP, suggesting the great potential of this material to achieve efficient MCT. Generally, |*Z*_in_/*Z*_0_| and *α* are used to evaluate the absorption and consume MV ability of the MV absorber, respectively.^[^ ^7a,b^ ^]^ When |*Z*_in_/*Z*_0_| value closing to 1.0, represents an excellent impedance matching for efficient MV absorption.^[^ ^15^ ^]^ Especially, the |*Z*_in_/*Z*_0_| value of CNT‐CuHHTP is 0.95, which is closer to 1 than that of CuHHTP (0.59) and CNT (0.07), indicating that CNT‐CuHHTP absorbed more incident MV than CuHHTP or CNT (Figure 3e). Besides, CNT‐CuHHTP with moderate *α* value is able to convert the absorbed MV into heat (Figure 3f). Although CNT has excessive *α* value, the incident MV is reflected on the surface of CNT due to the skin effect, resulting in poor MV absorption performance. Meanwhile, CuHHTP exhibits poor MV absorption performance due to the inappropriate |*Z*_in_/*Z*_0_| value and small *α* value. To sum up, owning to good impedance matching and moderate attenuation constant, the CNT‐CuHHTP has excellent microwaveocaloric performance.

In order to explore the MV attenuation mechanism of CNT‐CuHHTP, we further studied imaginary permittivity (*ε*″) and imaginary magnetic loss (*µ*″), which represent the ability to dissipate electromagnetic energy.^[^ ^16^ ^]^ Specifically, CNT‐CuHHTP with suitable dielectric loss (Figure 3g) and negligible magnetic loss (Figure 3h), indicate that the attenuation mechanism of CNT‐CuHHTP is dominated by dielectric loss. Generally, the dielectric loss capability of CNT‐CuHHTP is related to conduction loss and polarization relaxation. On the one hand, the high electrical conductivity of CNT‐CuHHTP endows them with efficient conduction loss. On the other hand, heterointerface polarization and dipole polarization are the main types of CNT‐CuHHTP polarization relaxation. And the polarization relaxation behaviors of CNT‐CuHHTP can be illustrated by the relationship between *ε*′ and *ε*″ curve in Figure 3i, the *ε*′–*ε*″ curve of CNT‐CuHHTP exhibits obvious Cole–Cole semicircles compared to the CNT (Figure 3i, inset) and CuHHTP (Figure S10, Supporting Information), demonstrating that several Debye relaxations processes occur in CNT‐CuHHTP. Further, through the electron hologram we observe a distinct charge distribution at the heterogeneous interface of CNT‐CuHHTP, demonstrating the intensively interfacial polarization in CNT‐CuHHTP (Figure 2j,k). These charge distributions caused by interface polarization are forced to re‐arrange orderly with the propagation direction of the alternating electric field to dissipate the MV energy. Besides, some dangling bonds on CNT and CuHHTP can quickly adjust their orientation under MV to adapt to the changing electric field direction, resulting in dipole polarization to dissipate MV energy. In conclusion, the efficient microwaveocaloric effect of CNT‐CuHHTP is attributed to the reasonable wave‐resistance matching and attenuation constant caused by multiple reflections, interface polarization, dielectric loss, and dipole polarization (Figure 3j).

### 2.4 Microwave Dynamics Performance and Mechanism of CNT‐CuHHTP

**Figure 4:**
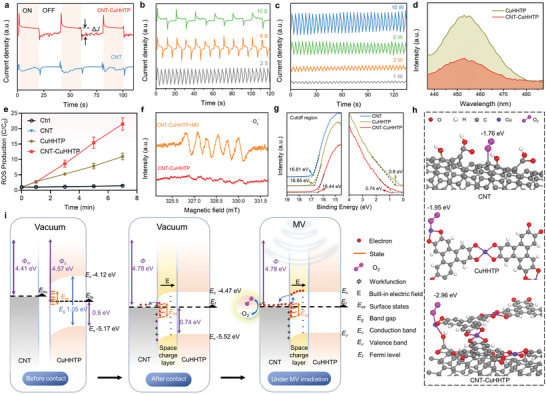
Microwave dynamics performance and mechanism of CNT‐CuHHTP. a) MV‐current responses of CNT and CNT‐CuHHTP under MV pulse irradiation. b) MV‐current responses of CNT‐CuHHTP under different MV pulse conditions. c) MV‐current responses of CNT‐CuHHTP under different MV powers. d) PL spectra of CuHHTP and CNT‐CuHHTP. e) ROS production with DCFH‐DA fluorescence probe. Data are presented as mean ± standard deviations from a representative experiment (*n*  =  3 independent samples). f) EPR measurements of ·O_2_^−^ of CNT‐CuHHTP before and after MV irradiation. g) UPS spectra of the CNT, CuHHTP, and CNT‐CuHHTP. h) Adsorption energy of CNT, CuHHTP, and CNT‐CuHHTP for oxygen. i) Schematical illustration for the mechanism of the ·O_2_^−^ production of CNT‐CuHHTP before and after MV irradiation.

Further, we tested the microwave dynamics performance of CNT‐CuHHTP. The transient MV‐current response was observed over three repeated cycles under 20 s MV pulse irradiation (a 20‐s pulse cycle consists of 20 s on, 20 s off). As shown in **Figure** **4**a, the CNT‐CuHHTP exhibits an obvious microwave response current, indicating that MV can excite the space charge separation in the CNT‐CuHHTP. In addition, the MV‐current density (Δ*J*) of CNT‐CuHHTP was obviously stronger than that of CNT, demonstrating the more effective charge transfer in the CNT‐CuHHTP heterointerface under MV irradiation. Notably, the MV‐current of CNT‐CuHHTP has shrilly front‐peak and back‐peak, which is consistent with the characteristics of surface states in the photocurrent,^[^ ^17^ ^]^ indicating CNT‐CuHHTP contains many surface states and defects, which are favorable for multi‐level transition of electron under the excitation of MV. In addition, the MV‐current density of CNT‐CuHHTP under different MV pulse conditions were almost similar, while the MV‐current also exhibited the obvious front‐peak and back‐peak (Figure 4b), indicating that the intensity of MV response current is independent of MV pulse conditions. Notably, the MV‐current intensity of CNT‐CuHHTP exhibits positively correlated with MV power, that is, the MV‐current becomes stronger as the MV power increases (Figure 4c). It indicates that we can significantly increase the amount of the excited electrons for ROS production by increasing the MV power. Besides, the photoluminescence (PL) spectra (Figure 4d) of CuHHTP and CNT‐CuHHTP revealed one absorption peak at ≈455 nm, and the PL signal of CNT‐CuHHTP is lower than that of CuHHTP indicated the heterostructure of CNT‐CuHHTP can efficiently reduce the recombination of electron−hole pairs, which are used to generate ROS. Therefore, CNT‐CuHHTP has higher ROS yield than CNT and CuHHTP under MV excitation (Figure 4e), which fully illustrates the enhanced contribution of CNT in microwave dynamics. And it is determined that the type of ROS is only ·O_2_^−^ (Figure 4f), not hydroxyl radicals (·OH) and singlet oxygen (^1^O_2_) (Figure S11, Supporting Information). Importantly, CNT‐CuHHTP hardly produces ·O_2_^−^ without MV, but a large amount of ·O_2_^−^ is produced under MV irradiation, which is beneficial for the specific responsiveness of MDT treatment.

According to the UV−vis diffuse reflectance spectra (Figure S12a, Supporting Information), the calculated plots (Figure S12b, Supporting Information) based on Kubelka−Munk function versus bandgap energy (*E*_g_) demonstrated that the bandgap of CuHHTP is 1.05 eV. Evidently, the energy of the MV (10^−3^ eV) is not enough to excite the bulk of CNT‐CuHHTP to generate charge carriers for ·O_2_^−^ (O_2_ + e^−^→·O_2_^−^). It is further demonstrated that the CNT‐CuHHTP has no bulk interband transition (direct transition from the valence band (*E*_v_) to the conduction band (*E*_c_) under MV irradiation), and the generation of carriers mainly depends on the surface states. Further, we determined the workfunction (*Φ*) of the samples by ultraviolet photoelectron spectroscopy (UPS). Specifically, the measured secondary electron cutoff energy of CNT, CuHHTP, and CNT‐CuHHTP were 16.81 eV, 16.65 eV, and 16.44 eV, respectively (Figure 4g). Accordingly, the *Φ* was calculated to be 4.41 eV, 4.57 eV, and 4.78 eV by subtracting the excitation energy of He I (21.22 eV). The distance between *E*_v_ and Fermi level (*E*_f_) of CuHHTP and CNT‐CuHHTP were 5.17 eV and 5.52 eV, respectively.

In order to better illustrate the mechanism of CNT‐CuHHTP to generate ·O_2_^−^ under MV excitation, we have summarized the oxygen adsorption energies for different sites of CNT, CuHHTP, and CNT‐CuHHTP. According to the principle of minimum energy, it can be seen that the C site of CNT is more likely to adsorb oxygen, and Cu (Cu—O_2_) site of CuHHTP and CNT‐CuHHTP is more likely to adsorb oxygen (Figure S13, Supporting Information). In addition, the adsorption energy of CNT‐CuHHTP (−2.96 eV) is lower than that of CNT (−1.76 eV) and CuHHTP (−1.95 eV) (Figure 4h), indicating that CNT‐CuHHTP is easier to adsorb oxygen than CNT and CuHHTP. With the local electric polarization, it is thermodynamically favorable for the Cu—O_2_ centers to strongly couple with O_2_ molecules, and thus beneficial for react with free electrons toward rapid formation of ·O_2_^−^ for antibacterial.

The MV‐dynamic mechanism of CNT‐CuHHTP is summarized in Figure 4i. Before CNT and CuHHTP contact, the surface energy band of CuHHTP bends downward due to the existence of surface states.^[^ ^18^ ^]^ After contact, the interaction of the energy band and interfacial structure promote MV‐dynamic effects. According to the pinning theory, the Fermi level of CNT will be pinned at the surface level of CuHHTP regardless of the work function of CNT.^[^ ^19^ ^]^ Thereby, a built‐in electric field (*E*) is formed, and the direction of the electric field is from the CNT side to the CuHHTP, that is, the CNT side has a positive charge and the CuHHTP side has a negative charge, which is consistent with the result of electron hologram charge distribution. Moreover, the built‐in electric field can drive electrons rapidly transferred from CuHHTP to CNT. As a result, under MV irradiation, MV can excite surface energy levels matching their energy to generate more MV‐excited electrons, which are transferred to CNT driven by a built‐in electric field to reduce recombination. These free electrons react with oxygen to generate ·O_2_^−^ for eradication of bacteria.

### 2.5 CNT‐CuHHTP Has Broad‐Spectrum Bactericidal Effect under MV Irradiation

**Figure 5:**
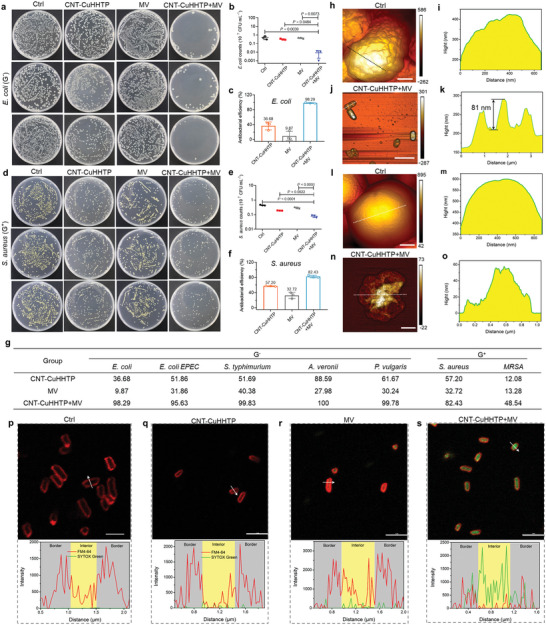
CNT‐CuHHTP has broad‐spectrum bactericidal effect under MV irradiation. Spread‐plate of a) *E. coli* and d) *S. aureus* treated with CNT‐CuHHTP with or without 7 min MW irradiation. b) *E. coli* and e) *S. aureus* strain counts calculated from spread‐plate assays. Antibacterial efficiency of CNT‐CuHHTP against c) *E. coli* and f) *S. aureus* with or without 7 min MW irradiation. g) Summary of antibacterial efficiency of different strains. h) AFM image and i) corresponding height profiles of *E. coli* without treated. Scale bar, 200 nm. j) AFM image and k) corresponding height profiles of *E. coli* after CNT‐CuHHTP+MV treated. Scale bar, 4 µm. l) AFM image and m) corresponding height profiles of *S. aureus* without treated. Scale bar, 210 nm. n) AFM image and o) corresponding height profiles of *S. aureus* after CNT‐CuHHTP+MV treated. Scale bar, 310 nm. p–s) Fluorescent localization (above) of *E. coli* under different conditions. The fluorescent dyes FM4‐64 to stain the OM (red), and SYTOX Green to show membrane permeabilization (green). Scale bar, 5 µm. Fluorescence intensity (below) along the white arrows in (p–s). Data are presented as mean ± standard deviations from a representative experiment (*n*  =  3 independent samples for b, c, e, f). *p*‐values were analyzed by one‐way ANOVA with Tukey's multiple comparisons post hoc test. Significance was defined as *p ≤* 0.05.

We tested the synergistic antibacterial effect of CNT‐CuHHTP with MV in vitro, including against typical Gram‐negative (G^−^) and Gram‐positive (G^+^) bacteria. It can be easily observed that the number of *E. coli* after CNT‐CuHHTP synergistic MV (CNT‐CuHHTP+MV) treatment for only 7 min was significantly reduced compared with other groups (**Figure** **5**a). Specifically, the number of *E. coli* (10^7^ CFU mL^−1^, Figure 5b) decreased from the original 0.4971 (Ctrl) to 0.0085 (CNT‐CuHHTP+MV), far lower than the CNT‐CuHHTP (0.3148) and MV (0.448) groups alone demonstrating that 98.29% *E. coli* were killed by CNT‐CuHHTP+MV (Figure 5c). Similarly, compared with Ctrl (not treated), CNT‐CuHHTP and MV groups, the treatment group of CNT‐CuHHTP+MV had the best antibacterial effect against *S. aureus* (Figure 5d), reducing the counts of *S. aureus* (10^7^ CFU mL^−1^, Figure 5e) from 0.4467 (Ctrl) to 0.0785 with 82.43% anti‐*S. aureus* efficiency (Figure 5f). Besides, we also tested four G^−^ bacteria including enteropathogenic *E. coli* (*E. coli* EPEC), *Salmonella typhimurium* (*S. typhimurium*), *Aeromonas veronii* (*A. veronii*), and *Proteus vulgaris* (*P. vulgaris*), and G^+^ bacteria methicillin‐resistant *S. aureus* (MRSA). In addition, the antibacterial effects of different treatment groups against different bacteria are shown in Figures S14–S18, Supporting Information, and the antibacterial results are summarized in Figure 5g. Specifically, for G^−^ bacteria the group of CNT‐CuHHTP had low antibacterial rates of 51.86% against *E. coli* EPEC, 51.69% against *S. typhimurium*, 88.59% against *A. veronii*, and 61.67% against *P. vulgaris*, respectively. Comparatively, the CNT‐CuHHTP+MV group showed higher bacterial‐killing efficiency against *E. coli* EPEC (95.63%), *S. typhimurium* (99.83%), *A. veronii* (100%), and *P. vulgaris* (99.78%). Similarly, for the antibacterial rate of CNT‐CuHHTP+MV group against G^+^ bacteria is higher than that of CuHHTP group, although its antibacterial rate against MRSA is only 48.54%. Notably, the antibacterial rate of CNT‐CuHHTP+MV group against *S. aureus* and MRSA can reach 99.93% and 96.77%, respectively, by increasing the MV power (Figure S19, Supporting Information). The CNT‐CuHHTP+MV group had the highest antibacterial rate, whether against G^−^ or G^+^ bacteria, it is worth noting that the antibacterial effect of CNT‐CuHHTP+MV against G^−^ bacteria (more than 95.6%) is higher than that against G^+^ bacteria (82.43% for *S. aureus* and 48.5% for MRSA) under the same antibacterial conditions.

We observed surface details of *E. coli* and *S. aureus* before (Ctrl) and after CNT‐CuHHTP+MV treatment by atomic force microscopy (AFM). Specifically, *E. coli* in the Ctrl group were rod‐like shape and plumply cell (Figure 5h). The complete profile presented by the corresponding height profile also proves its completeness (Figure 5i). Conversely, the morphology of *E. coli* in CNT‐CuHHTP+MV group showed severe collapses (Figure 5j) with a depth of 81 nm (Figure 5k). Similarly, the morphology of *S. aureus* was spherical and chubby (Figure 5l,m), but shrinkage and deformation in the CNT‐CuHHTP+MV group (Figure 5n,o). In addition, the SEM images of the four typical bacteria (Figure S20, Supporting Information) also showed a similar phenomenon: the Ctrl group showed a plump morphology, the CNT‐CuHHTP and MV groups both showed slight damage, and the CNT‐CuHHTP+MV group showed different degrees of collapse. Notably, the damage of CNT‐CuHHTP+MV to G^−^ bacteria is more obvious than that of G^+^ bacteria, which is consistent with the results of antibacterial rate. We speculated that this different antibacterial effect is related to the cell wall structure of these two types of bacteria. The cell membranes thickness of G^+^ bacteria is up to 15–30 nm due to the presence of a thick peptidoglycan layer,^[^ ^20^ ^]^ while the thickness of G^−^ bacteria is only 10–15 nm.^[^ ^21^ ^]^ The thick cell membrane of G^+^ bacteria weaken the destructive effect of high temperature on it, so the antibacterial efficiency of G^+^ is lower than that of G^−^ bacteria, even though exogenous ·O_2_^−^ is easier to penetrate the G^+^ membrane and enter the cell to play a bactericidal role.^[^ ^22^ ^]^ This speculation suggests that CNT‐CuHHTP+MV eliminates bacteria mainly by destroying the bacterial cell membranes.

Furthermore, taking *E. coli* as an example, we visualize the permeability of *E. coli* membranes using the membrane dye FM4‐64 and the nucleic acid stain SYTOX Green. Generally, SYTOX Green will not pass through the intact cell membrane, and only when the bacterial membrane is damaged, SYTOX Green will enter the cell and cause cells to display green fluorescence. *E. coli* in the Ctrl group (without treatment) showed the outline of the bacterial membrane without the green fluorescent signal (Figure 5p). The CNT‐CuHHTP (Figure 5q) and MV (Figure 5r) groups showed strong red signals and weak green signals indicating that CNT‐CuHHTP and MV had a slight damaging effect on *E. coli* cell membranes. Conversely, in the CNT‐CuHHTP+MV group (Figure 5s), a clear intracellular green signal was displayed, the linear scan results showed good colocalization, and the green signal intensity was higher than other groups indicating that the cell membrane of *E. coli* was seriously damaged and leads to its death.

In addition, the normal saline solution of CNT‐CuHHTP (1 mg mL^−1^) can release 11.56 ppm copper ion in 48 h. After MV irradiation for 7 min, the concentration of copper ion can reach 10.18 ppm (Figure S21, Supporting Information). This shows that MV irradiation of CNT‐CuHHTP will release a small amount of copper ions, which is conducive to the elimination of bacteria.

To sum up, these results demonstrate that CNT‐CuHHTP generated ·O_2_^−^ and microwaveocaloric under MV irradiation,destroy the bacterial cell membrane structure, and conducive to the entry of copper ions into the cell, thus causing the efflux of intracellular substances and the collapse of the cytoskeleton, eventually leading to bacterial death.

### 2.6 Safety Evaluation of CNT‐CuHHTP

We investigated the cytocompatibility of CNT‐CuHHTP by observing the morphological changes and cell viability of cells after co‐culture with CNT‐CuHHTP. Specifically, after MC3T3‐E1 was co‐cultured with CNT‐CuHHTP for 3 days (Figure S22, Supporting Information), the cells were spindle‐shaped, conical, or cubic, with obvious nuclei, and short protrusions on the cell surface were connected to adjacent cells, which had no significant difference from the cell morphology of the control group (Ctrl). Likewise, CNT‐CuHHTP did not have a significant effect on the morphology of A549 cells. In addition, the cell viabilities of MC3T3‐E1 were concentration‐dependent, and the cell viability increased with the decrease of CNT‐CuHHTP concentration. The cell viabilities of MC3T3‐E1 remained above 94% even with CNT‐CuHHTP concentration of 1 mg mL^−1^ (Figure S23, Supporting Information). These results demonstrate the excellent cytocompatibility of CNT‐CuHHTP in vitro.

Moreover, we have evaluated the effect of CNT‐CuHHTP+MV (consistent with the irradiation conditions in animal experiments) on the viability of A549. As show in Figure S24, Supporting Information, after CNT‐CuHHTP+MV treatment for one day, the morphology of A549 cells changed from a polygonal shape with filamentous pseudopodia to a fusiform antenna that shortened and shrank, and the cells shrank into a spherical structure. Continue to culture to the third day, the cell morphology returned to normal, and the number of cells increased. These results indicate that cell proliferation will be inhibited after CNT‐CuHHTP+MV treatment, but after a short period of recovery, the cells can gradually recover and begin to proliferate.

**Figure 6:**
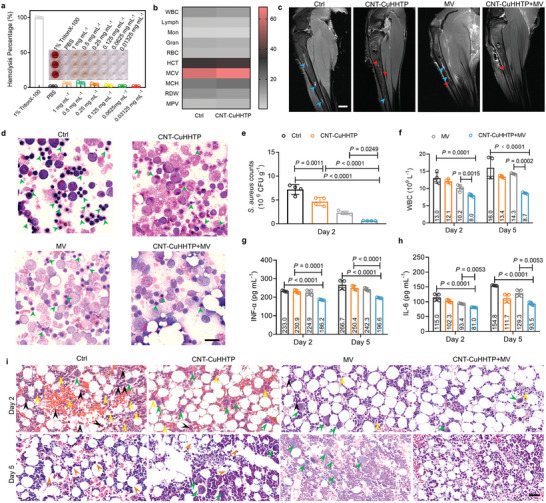
Safety evaluation of CNT‐CuHHTP and therapeutic effect on *S. aureus*‐infected osteomyelitis. a) Hemolytic efficiency of different concentrations of CNT‐CuHHTP. Inset: The representative image demonstrating the varying degrees of hemolysis. *n*  =  3 biologically independent samples. b) Blood routine tests of rabbits after injection of normal saline (Ctrl) or CNT‐CuHHTP at 5 days post‐injection. WBC, white blood cells; Lymph, number of lymphocytes; Mon, monocyte; Gran, granulocyte; RBC, red blood cells; HCT, hematocrit; MCV, mean red blood cell volume; MCH, mean erythrocyte hemoglobin; RDW, red blood cell distribution width. MPV, mean platelet volume. c) MRI after 5 days treatment. Scale bars = 1 cm. d) Wright‐stained images of bone marrow tissue after 5 days of different treatments. Scale bars = 20 µm. e) The *S. aureus* counts in the bone marrow after 2 days with different treatments. *p*‐values were analyzed by one‐way ANOVA with Tukey's multiple comparisons post hoc test for (e). f) Amount of WBC at 2 and 5 days in blood. g) INF‐*α* levels and h) IL‐6 at 2 and 5 days in blood. Data are presented as mean ± standard deviations from a representative experiment (*n*  =  4 independent samples for (e–h)). *p*‐values were analyzed by two‐way ANOVA with Tukey's multiple comparisons post hoc test for (f–h). i) H&E staining images of bone marrow. Scale bars = 20 µm. Significance was defined as *p ≤* 0.05.

Besides, CNT‐CuHHTP has good blood compatibility and in vivo safety. Specifically, there was no obvious hemolysis after co‐culture of CNT‐CuHHTP with rabbit erythrocytes (**Figure** **6**a, inset). The hemolysis rate of CNT‐CuHHTP (1 mg mL^−1^) was only 6.3% (Figure 6a), which was much lower than that of the positive control group (1% TritonX‐100). Meanwhile, after injection of CNT‐CuHHTP, there was no abnormality in the corresponding indicators of blood routine (WBC, Lymph, Mon, Gran, RBC, HCT, MCV, MCH, RDW, and MPV) in rabbits (Figure 6b). There was no significant difference in the hepatic function (Figure S25a,b, Supporting Information) and renal function (Figure S25c,d, Supporting Information) between the Ctrl and CNT‐CuHHTP group at the given dose. Hematoxylin and eosin (H&E) sections (Figure S26, Supporting Information) of major organs (heart, liver, spleen, lung, kidney) show that CNT‐CuHHTP has no organ toxicity, further demonstrating the excellent in vivo safety of CNT‐CuHHTP.

### 2.7 Eradication of Osteomyelitis

The surgical procedure for modeling rabbit osteomyelitis is shown in Figure S27, Supporting Information, which mainly includes disinfection of the skin surface, drilling of the tibia, injection of bacteria, bone wax sealing, and suture of tissue. The treatment process of osteomyelitis, taking the CNT‐CuHHTP+MV group as an example, was to add CNT‐CuHHTP after adding the bacterial solution, suture the tissue, and then irradiate MV for 7 min. The details are described in Section 4. The temperature distribution of rabbit tissue before and after CNT‐CuHHTP+MV treatment is shown in Figure S28, Supporting Information. The temperature rises from 28 to 42 °C, which will not cause serious tissue damage. The MRI image (Figure 6c) exhibited the inflammation of the bacterial‐infected bone marrow site is hyperintense (indicated by blue arrows) and accompanied by bone defects (indicated by red arrows). After CNT‐CuHHTP+MV treatment, the inflammatory signals were attenuated, and bone defect symptoms were reduced. According to the Wright's staining of bone marrow (Figure 6d), it can be seen that the Ctrl, CNT‐CuHHTP, and MV group have different degrees of red blood cell damage, accompanied by a large number of lymphocytes (indicated by green arrows). In contrast, the morphology of erythrocytes was complete and the number of inflammatory cells was less in the CNT‐CuHHTP+MV group. In addition, the number of *S. aureus* (10^6^ CFU g^−1^) in the bone marrow in the CNT‐CuHHTP+MV group was 0.619, which was also the least compared to the Ctrl (7.181), CNT‐CuHHTP (4.635), and MV (2.277) groups (Figure 6e). These results demonstrate that CNT‐CuHHTP+MV has an excellent antibacterial effect in vivo.

Besides, we analyzed the blood samples and bone marrow tissue histopathological section of the rabbit after treatment. Specifically, after 2 days of treatment, the number of WBC (Figure 6f) in the treatment group showed a decreasing trend, and the CNT‐CuHHTP+MV group decreased to 8.0 (10^9^ L^−1^) reached the normal level. A similar trend emerged at after 5 days of treatment. To evaluate the inflammatory response of different treatment groups the levels of TNF‐*α* and IL‐6 were measured. The CNT‐CuHHTP+MV group had the lowest concentrations of TNF‐*α* (Figure 6g) and IL‐6 (Figure 6h) whether on the second day (Day 2) or the fifth day (Day 5) after treatment, indicating that the treatment of CNT‐CuHHTP+MV did not cause obvious inflammatory response, on the contrary, the other groups had caused a significant inflammatory storm.

Further, HE sections of bone marrow tissue (Figure 6i) showed that the CNT‐CuHHTP+MV group had fewer inflammatory cells at the early stage of treatment (Day 2), while the Ctrl group had more multinucleated giant cells (indicated by green arrows), neutrophils (indicated by back arrows), and lymphocytes (indicated by yellow arrows) in the bone marrow. The presence of large numbers of inflammatory cells indicates a serious infection in the bone marrow. At Day 5, the bone marrow of the Ctrl group showed a large number of adipocyte rupture (indicated by orange arrows), and the degree of rupture was less in the treatment group, especially in the group of CNT‐CuHHTP+MV. Systemic infection caused by osteomyelitis can lead to lesions of the organs. The pathological sections of the main organs in different groups are shown in Figure S29, Supporting Information. *S. aureus* infection causes punctate necrosis of cardiomyocytes (indicated by red arrows), diffuses distribution of inflammatory cells in the liver and spleen (indicated by yellow), and alveolar epithelial hyperplasia with red mucus (indicated by black arrows). These symptoms almost disappeared in group of CNT‐CuHHTP+MV, demonstrating the excellent antibacterial properties of MCT and MDT in vivo. Notably, the improvement effect of CNT‐CuHHTP+MV on osteomyelitis is comparable to that of traditional antibiotics gentamicin (Gent) (Figure S30, Supporting Information).

## 3 Conclusion

CNT confined the epitaxial growth of CuHHTP, resulting in the formation of ultra‐small CuHHTP growing along the CNT skeleton, accompanied by abundant surface states which were mainly derived from the surface/interface defects (surface steps, unsaturated bonds and lattice mismatch) of CNT‐CuHHTP. CNT‐CuHHTP can efficiently kill five Gram‐negative bacteria (*E. coli*, *E. coli* EPEC, *S. typhimurium*, *A. veronii*, and *P. vulgaris*) and two Gram‐positive bacteria (*S. aureus* and MRSA) with only 7 min of MV irradiation. Its robust broad‐spectrum antibacterial effectiveness is mainly attributed to the destruction of bacterial membranes caused by microwaveocaloric and microwave dynamics effects (·O_2_^−^). Specifically, the efficient microwaveocaloric effect of CNT‐CuHHTP is own to the reasonable wave‐resistance matching and attenuation constant caused by multiple reflections, interface polarization, dielectric loss and dipole polarization. Besides, under microwave irradiation, CNT‐CuHHTP generates excited electrons through the surface energy level, and the built‐in electric field accelerates the transfer of excited electrons to CNT and promotes the generation of ·O_2_^−^. The in vivo results confirm that this antibiotic‐free microwave therapy can cure osteomyelitis by reducing bacterial counts with topical treatments. This platform may bring further insight and understanding of deep tissue infection diseases treatment through antibiotic‐free microwave therapy.

## 4 Experimental Section

### Materials

Multi‐walled CNT was purchased from Aiweixin Chemical Technology Co., Ltd. (Tianjin, China). Copper nitrate trihydrate (Cu (NO_3_)_2_·3H_2_O) was purchased from Aladdin. HHTP was purchased from Meryer (China). SYTOX Green Ready Flow Reagent (R37168), and FM 4–64 (T13320) were purchased from Thermo Fisher scientific (USA). The rabbit Interleukin‐6 ELISA Kit (CK‐E72064) and rabbit tumor necrosis factor alpha (TNF‐*α*) ELISA Kit (CK‐E72292) were purchased from Nanjing Herbal Source Biotechnology Co., Ltd. (China).

### Synthesis of CuHHTP and CNT‐CuHHTP

In a typical synthesis, the HHTP ligand (30 mg) was dispersed by ultrasound in 4 mL water, and the aqueous solution (4 mL) of copper acetate (30 mg) and CNT (10 mg) mixed by ultrasound was added slowly. The mixture was sonicated for 15 min and the blue solution was obtained. Subsequently, the blue solution was transferred to the glass sample bottle and heated at 85 °C for 24 h. The CNT‐CuHHTP was obtained after centrifuged and washed with water and acetone, respectively. The obtained CNT‐CuHHTP was dried under vacuum. The same preparation process was used to obtain CuHHTP, except that no CNT was added during the reaction process.

### Characterization of CNT‐CuHHTP

The TEM image and elemental mapping images of CNT‐CuHHTP were observed using TEM (JEM‐2100F). The HAADF‐STEM image was observed using JEOL JEM‐ARM200F. The HRTEM images and off‐axis electron holography measurements were performed using image spherical aberration corrected transmission electron microscopy (Image Cs‐Corrected TEM) (FEI‐Themis Z). The phase reconstruction was processed by a dedicated script in the Digital Micrograph software package (Gatan Inc.) based on the fast Fourier transform (FFT) algorithm on the image spherical aberration corrected transmission electron microscopy (Image Cs‐Corrected TEM) (FEI‐Themis Z). The crystal structures and surface compositions were collected with XRD (DX‐2700BH, Haoyuan Instrument Co., Ltd.) and XPS (Axis Supra, Kratos). Cu K‐edge XAFS analyses were performed with Si (111) crystal monochromators at the BL14W beamline at the Shanghai Synchrotron Radiation Facility (SSRF) (Shanghai, China). Before the analysis at the beamline, samples were placed into aluminum sample holders and sealed using Kapton tape film. The XAFS spectra were recorded at room temperature using a 4‐channel Silicon Drift Detector (SDD) Bruker 5040. The spectra was processed and analyzed by the software codes Athena.

### Microwaveocaloric Effect Measurements

The saline solution of CNT‐CuHHTP (1 mg mL^−1^) was dispersed by ultrasound in a 2 mL Eppendorf (EP) tube. Then the EP tube with CNT‐CuHHTP solution (1 mL) was irradiated by microwave therapy instrument (Schneider Medical Equipment Co., Ltd., China) at 2.45 GHz for 7 min. The temperature of the solution was recorded every minute using an FLIR thermal camera (FLIR E64501, Estonia). Similarly, the microwaveocaloric temperatures of CNT and CuHHTP in saline solution were also collected by this method.

Subsequently, infrared thermal images were also obtained by FLIR thermal camera. The CNT‐CuHHTP solution (1 mL, 1 mg mL^−1^) in 2 mL EP tube was placed on pork with different thicknesses (0, 5, 10, and 25 mm) and applied MV for 7 min across the biological tissues.

### Electromagnetic Measurements

First, CNT, CuHHTP, and CNT‐CuHHTP were uniformly mixing with paraffin matrix according to a same mass fraction of 40%, respectively. And compacted the mixtures into columnar ring of with a 7.00‐mm outer diameter and a 3.04‐mm inner diameter. These columnar rings were measured the electromagnetic parameters using a vector network analyzer (Agilent PNA‐N5244A, USA). The MV absorption performances of CNT, CuHHTP, and CNT‐CuHHTP were evaluated by calculating the RL based on the transmission line theory.

(2) $$z_{\mathrm{in}}=z_{0}\;\sqrt{\frac{\mu_{r}}{\varepsilon_{r}}}\tanh\left[j\left(\frac{2\;f\pi t}{c}\right)\right]\sqrt{\mu_{r}\varepsilon_{r}}$$

(3) $$\mathrm{RL}\;=\;20\;\log\left|\frac{z_{\mathrm{in}}-z_{o}}{z_{\mathrm{in}}+z_{0}}\right|$$

where *Z*_in_ is the input impedance at the absorber surface, *Z*_0_ is the impedance of the air, *f* is the MV frequency, *t* is the thickness of the absorber, and *c* is the velocity of light in free space.

The attenuation constant *α* was calculated as the following Equation (4):

(4) $$\begin{array}{ccc} \alpha & = & \frac{\sqrt{2}\pi f}{c}\\ & & \times \sqrt{\left(\mu^{\prime \prime }\varepsilon^{\prime \prime }-\mu^{\prime }\varepsilon^{\prime }\right)+\sqrt{{\left(\mu^{\prime \prime }\varepsilon^{\prime \prime }-\mu^{\prime }\varepsilon^{\prime }\right)}^{2}+{\left(\mu^{\prime }\varepsilon^{\prime \prime }+\mu^{\prime \prime }\varepsilon^{\prime }\right)}^{2}}} \end{array}$$

### Microwave Dynamics Evaluation

MV‐electrochemical measurements were based on an electrochemical workstation (CHI 660E) equipped with 2.45 GHz microwave source. Moreover, a three‐electrode system in quartz glass cell in Na_2_SO_4_ (0.5 m) aqueous solution was built using a Pt plate as counter electrode, an Ag/AgCl electrode as reference electrode, and ITO conductive glasses (1 cm × 1 cm) with CNT or CNT‐CuHHTP as working electrode. UV–vis diffuse reflectance spectra were recorded by UV–vis spectrophotometer (UV‐2700, Shimadzu). Photoluminescence spectrofluorometer (Fluorolog‐3, Horiba Jobin Yvon) was conducted with an excitation wavelength of 400 nm.

The ROS amounts of the saline solutions of CNT, CuHHTP, and CNT‐CuHHTP (1 mg mL^−1^) under continuous MV irradiate were obtained using a ROS Assay Kit (S0033S, Beyotime). Additionally,·O_2_^−^ and ·OH were detect using 5,5‐dimethyl‐1‐pyrroline‐*N*‐oxide (DMPO, Aladdin) as the trapping agent measured by electron paramagnetic resonance (EPR, JES‐FA200, JEOL) spectrometer. Besides, ^1^O_2_ was using 2,2,6,6‐tetramethylpiperidine (TEMP, Aladdin) as the trapping agent.

### Theoretical Calculations

All of spin‐polarized calculations based on density functional theory (DFT) were performed by utilizing DMol3 package. The generalized gradient approximation (GGA) in the Perdew–Burke–Ernzerhof form and Semicore Pseudopotential method (DSPP) with the double numerical basis sets plus the polarization functional (DNP) were adopted. The Brillouin zones were sampled by using Monkhorst–Pack scheme with a 1×1×1k‐point grid and the double numerical basis sets plus the polarization functional (DNP) were adopted. A DFT‐D correction with Grimme scheme was used to account for the dispersion interaction. The SCF convergence for each electronic energy was set as 1.0 × 10^−6^ Ha, and the geometry optimization convergence criteria were set up as follows: 1.0 × 10^−5^ Ha for energy, 0.0004 Ha Å^−1^ for force, and 0.001 Å for displacement, respectively. Finally, the adsorption energies (*E*_ads_) are calculated as *E*_ads_ = *E*_ad/sub_ − *E*_ad_ – *E*_sub_, where *E*_ad/sub_, *E*_ad_, and *E*_sub_ are the optimized adsorbate/substrate system, the adsorbate in the structure and the clean substrate, respectively.

### Antibacterial Test

The antibacterial test of CNT‐CuHHTP with or without MV against *E. coli* (ATCC 8099), *E. coli* EPEC (CICC 10663), *S. typhimurium* (CCTCC PB 2019001), *A. veronii* (ATCC 35624), *P. vulgaris* (CCTCC AB 91103), *S. aureus* (ATCC 25923), and MRSA (CCTCC 16465) were quantitatively using the spread plate method. The bacterial suspensions were diluted to about 10^7^ CFU mL^−1^ for antibacterial experiments. Subsequently, the diluted bacterial suspensions were incubated with physiological saline (Ctrl) and CNT‐CuHHTP (1 mg mL^−1^) in 2 mL EP tubes with and without MV irradiation for 7 min. After different treatments, the appropriate diluted bacterial solution was smeared on LB agar plate by coater and cultured at 37 °C. The colonies were counted after cultured 16–20 h to calculate the antibacterial ratio, which was then assessed using Equation (5):

(5) $$\mathrm{Antibacterial}\;\mathrm{ratio}\;(\%)\;=\frac{A-B}{A}\;100\%$$

where *A* and *B* represent the numbers of bacteria in the control group (Ctrl) and experimental group, respectively.

The morphologies of *E. coli* and *S. aureus* before and after treatment with CNT‐CuHHTP+MV were measured by atomic force microscopy (Agilent 5500, Bruker Dimension Icon). In addition, the morphologies of the other bacteria (*E. coli* EPEC, *S. typhimurium*, *S. aureus*, and MRSA) interacting with CNT‐CuHHTP with or without MV were evaluated using SEM (S‐4800, Hitachi) after fixation and dehydration.^[^ ^23^ ^]^

### Morphological Staining of *E. coli*

The *E. coli* stock solution was washed three times by centrifugation with normal saline, and then diluted to obtain a suspension with a concentration of 10^7^ CFU mL^−1^. After different treatments, SYTOX Green (5 µm) was added and incubated for 4 min at room temperature. Then, FM4‐64 (5 ppm) was added and incubated for 1 min (on ice). Subsequently, the two stained cultures were centrifuged at 6000 × *g* for 30 s (4 °C) and resuspended in normal saline (1/10 volume). The resuspension solution (5 µL) was taken on a glass slide and covered with a coverslip to be photographed by a laser scanning confocal microscope (A1R+, Nikon).

### In Vitro Cytotoxicity Evaluation

The MC3T3‐E1 (ATCC CRL‐2593) and A549 (ATCC CCL‐185) were cultured in Dulbecco's modified eagle medium and minimum essential medium alpha (*α*‐MEM) respectively, including supplemented with 10% v/v fetal bovine serum, 1% amphotericin, and 1% penicillin–streptomycin solution, and incubated at 37 °C containing with 5% CO_2_.

### MTT Assay of MC3T3‐E1

The various concentrations of CNT‐CuHHTP (1 mg mL^−1^, 512 ppm, 256 ppm, and 128 ppm) were co‐cultured with MC3T3‐E1 cells (10^5^ cells/well). The Ctrl group was used the same volume of physiological saline instead of CNT‐CuHHTP. Subsequently, the cell viability was obtained using a MTT (3‐(4,5‐dimethylthiazol‐2‐yl)‐2,5‐diphenyltetrazolium bromide) cell proliferation and cytotoxicity assay kit (C0009S, Beyotime).

For the fluorescence morphology, MC3T3‐E1 or A549 cells were first incubated in six‐well plates for 24 h and further incubated with/without CNT‐CuHHTP (1 mg mL^−1^). The treated cells were cultured for three days. After incubation, the samples were washed with PBS, soaked in formaldehyde (4%) for 10 min, and rinsed with sterile PBS. These samples were then stained with isothiocyanate‐phalloidin (FITC) for 30 min and 4,6‐Diamidino‐2‐phenylindole (DAPI) for 20 s in the dark. Subsequently, the morphology of samples after rinsing with PBS were photographed using laser scanning confocal microscopy (A1R+, Nikon). In the experiment of evaluating the effect of CNT‐CuHHTP+MV treatment on A549, the MV (7 min, 0.1 W cm^−2^) was irradiated after adding CNT‐CuHHTP, and the subsequent staining method was consistent with the above steps.

Evaluation hemolysis of CNT‐CuHHTP. 5 mL of New Zealand rabbit blood was diluted with PBS (50 mL) centrifuged (176 × *g*) for 5 min to obtain red blood cells (RBCs). Subsequently, the collected RBCs were resuspended using 10 mL PBS. Different concentrations of CNT‐CuHHTP were incubated with the diluted RBCs for four hours at 37 °C. After co‐cultured and centrifugated, the absorbance of the supernatant was measured at 405 nm. The positive control (Hemolytic efficiency was 100%) was used with 1% TritonX‐100 instead of CNT‐CuHHTP. The hemolysis percentage was calculated by comparing the absorbance values of these samples with the positive control.

### In Vivo Safety

After drilled the hind legs of male New Zealand rabbits, 200 µL of CNT‐CuHHTP (5 mg mL^−1^) was injected in situ. After five days of operation, the blood routine, renal function, and hepatic function were measured. And histological analysis of major organs (heart, liver, spleen, lung, and kidney) was carried out.

### Rabbit Osteomyelitis Model

The study was carried out in accordance with the Guide for the Care and Use of Laboratory Animals of the National Institutes of Health. The ethical aspects of the animal experiment were approved by Yi Shengyuan Gene Technology (Tianjin) Co., Ltd. (Approval No. YSY‐DWLL‐2021034). Male New Zealand rabbits (6–8 weeks old, 2.0–2.5 kg in weight) were randomly divided into four groups: the group of physiological saline (Ctrl), the treatment group of CNT‐CuHHTP (CNT‐CuHHTP), the treatment group of MV, the collaborative treatment group of CNT‐CuHHTP and MV (CNT‐CuHHTP +MV), and positive control antibiotic group (Gent).

The rabbits were raised for 3 days in a 25 ± 2 °C and 60–70% (humidity) conditions. In brief, they were anaesthetized with an intramuscular injection of pentobarbital (30 mg kg^−1^) prior to surgery. Then, hind legs were shaved and disinfected (iodophor) at the tibia and femur. After that, a hole was drilled in the tibial plateau by medical electric drill with a drill bit diameter of 2 mm. Subsequently, the legs were injected with 10^6^ CFU (50 µL) of *S. aureus* suspension via this hole to establish the *S. aureus*‐infected osteomyelitis model. Physiological saline, Gent or CNT‐CuHHTP was added for different treatment options. Finally, the holes were sealed with bone wax and the muscles and skin were sutured. The rabbits were subjected to different treatments: The Ctrl group was injected with physiological saline (200 µL). The CNT‐CuHHTP group was injected with CNT‐CuHHTP (200 µL, 5 mg mL^−1^). The Gent group was injected with Gent (3 mg kg^−1^). The MV group was injected with physiological saline (200 µL), and then irradiated MV (2.45 GHz, 0.1 W cm^−2^) for 7 min; and the CNT‐CuHHTP + MV group was injected with 200 µL of CNT‐CuHHTP (5 mg mL^−1^) and irradiated with MV for 7 min.

After 5 days of treatment, the hind legs of the infected rabbits were examined by Animal Magnetic Resonance Imaging Platform (BioSpec 94/30 USR, Bruker) At a preset time, the blood of the rabbits were collected for INF‐*α*, IL‐6, and WBC tests. Their bone marrow was collected for Wright's staining (Day 5) and H&E staining (Day 2 and 5). After five days of treatment, the major organs were stained with H&E. The bone marrow was quantitatively diluted to counted the number of *S. aureus* colonies by spread‐plate method.

### Statistical Analysis

Each experiment contained at least three parallel samples and presented as mean ± standard deviation. A *t*‐test was used for two‐group comparison and one‐ or two‐way analysis of variance (ANOVA) for multiple comparisons (GraphPad Prism 8.0 software). Significant differences are indicated by *p*‐values of less than 0.05. n.s. presents no significant difference.

## Conflict of Interest

The authors declare no conflict of interest.

## Supporting information

Supporting Information

## Data Availability

The data that support the findings of this study are available from the corresponding author upon reasonable request.

## References

[1] E. A. Masters , R. P. Trombetta , K. L. de Mesy Bentley , B. F. Boyce , A. L. Gill , S. R. Gill , K. Nishitani , M. Ishikawa , Y. Morita , H. Ito , S. N. Bello‐Irizarry , M. Ninomiya , J. D. Brodell , C. C. Lee , S. P. Hao , I. Oh , C. Xie , H. A. Awad , J. L. Daiss , J. R. Owen , S. L. Kates , E. M. Schwarz , G. Muthukrishnan , Bone Res. 2019, 7, 20. 31646012 10.1038/s41413-019-0061-z PMC6804538

[2] L. Urish Kenneth , E. Cassat James , M. Ottemann Karen , Infect. Immun. 2020, 88, e00932. 32094258 10.1128/IAI.00932-19 PMC7309607

[3] B. D. Gimza , J. E. Cassat , Front. Immunol. 2021, 12, 638085. 33643322 10.3389/fimmu.2021.638085 PMC7907425

[4] a) P. Zhao , Z. Jin , Q. Chen , T. Yang , D. Chen , J. Meng , X. Lu , Z. Gu , Q. He , Nat. Commun. 2018, 9, 4241; 30315173 10.1038/s41467-018-06630-2 PMC6185976

[5] J. Qi , W. Li , K. Lu , F. Jin , D. Liu , X. Xu , X. Wang , X. Kang , W. Wang , G. Shu , F. Han , X. Ying , J. You , J. Ji , Y. Du , Nano Lett. 2019, 19, 4949. 31286769 10.1021/acs.nanolett.9b01061

[6] M. Qin , L. Zhang , H. Wu , Adv. Sci. 2022, 9, 2105553. 10.1002/advs.202105553 PMC8981909 35128836

[7] a) Y. Qiao , X. Liu , B. Li , Y. Han , Y. Zheng , K. W. K. Yeung , C. Li , Z. Cui , Y. Liang , Z. Li , S. Zhu , X. Wang , S. Wu , Nat. Commun. 2020, 11, 4446; 32895387 10.1038/s41467-020-18268-0 PMC7477539

[8] Z. Sang , J. Liu , X. Zhang , L. Yin , F. Hou , J. Liang , ACS Nano 2023, 17, 3077. 36688450 10.1021/acsnano.2c11974

[9] J.‐D. Yi , R. Xie , Z.‐L. Xie , G.‐L. Chai , T.‐F. Liu , R.‐P. Chen , Y.‐B. Huang , R. Cao , Angew. Chem., Int. Ed. 2020, 59, 23641. 10.1002/anie.202010601 32926542

[10] W. Cheng , H. Zhang , D. Luan , W. L. Xiong , Sci. Adv. 2021, 7, eabg2580. 33910899 10.1126/sciadv.abg2580 PMC8081372

[11] a) N. E. Leadbeater , Angew. Chem., Int. Ed. 2006, 45, 1677;

[12] a) N. Fukui , R. Hobara , A. Takayama , R. Akiyama , T. Hirahara , S. Hasegawa , Phys. Rev. B 2020, 102, 115418;

[13] X. Xu , Y. Liu , J. Wang , D. Isheim , V. P. Dravid , C. Phatak , S. M. Haile , Nat. Mater. 2020, 19, 887. 32284599 10.1038/s41563-020-0656-1

[14] H. Sun , R. Che , X. You , Y. Jiang , Z. Yang , J. Deng , L. Qiu , H. Peng , Adv. Mater. 2014, 26, 8120. 25338951 10.1002/adma.201403735

[15] Y. Li , X. Liu , X. Nie , W. Yang , Y. Wang , R. Yu , J. Shui , Adv. Funct. Mater. 2019, 29, 1807624.

[16] M. Huang , L. Wang , K. Pei , W. You , X. Yu , Z. Wu , R. Che , Small 2020, 16, 2000158. 10.1002/smll.202000158 32182407

[17] Q. Zhang , H. Guo , Z. Feng , L. Lin , J. Zhou , Z. Lin , Electrochim. Acta 2010, 55, 4889.

[18] S. Lin , R. Shen , T. Yao , Y. Lu , S. Feng , Z. Hao , H. Zheng , Y. Yan , E. Li , Adv. Sci. 2019, 6, 1901925. 10.1002/advs.201901925 PMC6918112 31871865

[19] R. B. Darling , Phys. Rev. B 1991, 43, 4071. 10.1103/physrevb.43.4071 9997756

[20] W. Vollmer , D. Blanot , M. A. De Pedro , FEMS Microbiol. Rev. 2008, 32, 149. 18194336 10.1111/j.1574-6976.2007.00094.x

[21] A. Mai‐Prochnow , M. Clauson , J. Hong , A. B. Murphy , Sci. Rep. 2016, 6, 38610. 27934958 10.1038/srep38610 PMC5146927

[22] J.‐H. Cheng , X. Lv , Y. Pan , D.‐W. Sun , Trends Food Sci. Technol. 2020, 103, 239.

[23] L. Tan , J. Fu , F. Feng , X. Liu , Z. Cui , B. Li , Y. Han , Y. Zheng , K. Yeung Kelvin Wai , Z. Li , S. Zhu , Y. Liang , X. Feng , X. Wang , S. Wu , Sci. Adv. 2020, 6, eaba5723. 33188012 10.1126/sciadv.aba5723 PMC10763977

