# Supplementary material for: Enhancing Microwave Dynamic Effects via Surface States of Ultrasmall 2D MOF Triggered by Interface Confinement for Antibiotics‐Free Therapy
Source: Adv Sci (Weinh). 2023 May 18;10(21):2300084. doi: 10.1002/advs.202300084 (PMC10375132; doi:10.1002/advs.202300084)
Supplement: Supplementary file 1 — Supporting Information [file ADVS-10-2300084-s001.pdf]

ADVANCED SCIENCE
Open Access

Supporting Information

for *Adv. Sci.*, DOI 10.1002/advs.202300084

Enhancing Microwave Dynamic Effects via Surface States of Ultrasmall 2D MOF Triggered by Interface Confinement for Antibiotics-Free Therapy

Yuqian Qiao, Shuilin Wu, Yufeng Zheng, Chaofeng Wang, Zhaoyang Li, Yu Zhang, Shengli Zhu, Hui Jiang, Zhenduo Cui and Xiangmei Liu**

Supporting Information

Enhancing microwave dynamic effects via surface states of ultrasmall 2D MOF triggered by interface confinement for antibiotics-free therapy

Yuqian Qiao, Shuilin Wu, Yufeng Zheng, Chaofeng Wang, Zhaoyang Li, Yu Zhang, Shengli Zhu, Hui Jiang, Zhenduo Cui, Xiangmei Liu**

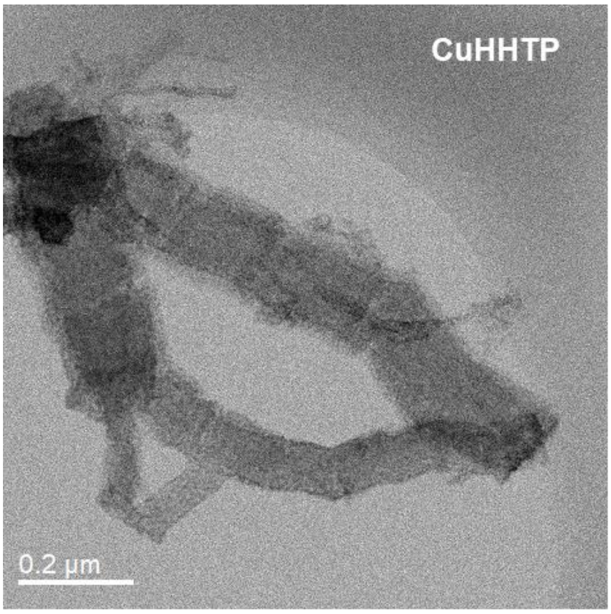

Figure S1 Typical bright-field TEM image of CuHHTP.

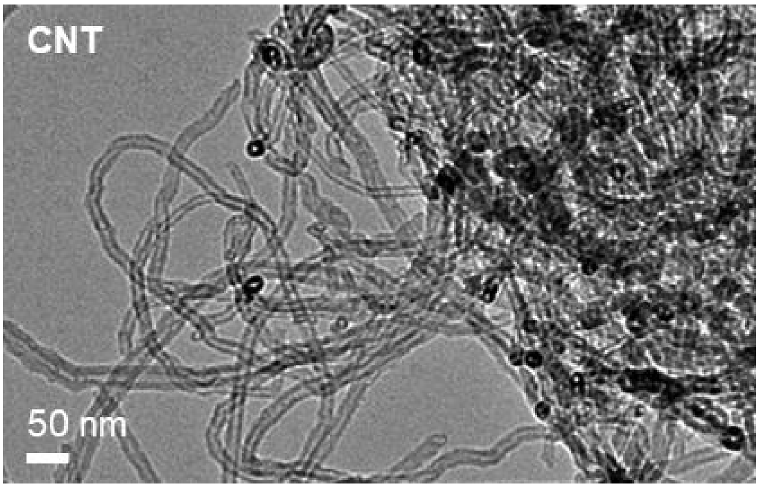

Figure S2 Typical TEM image of CNT.

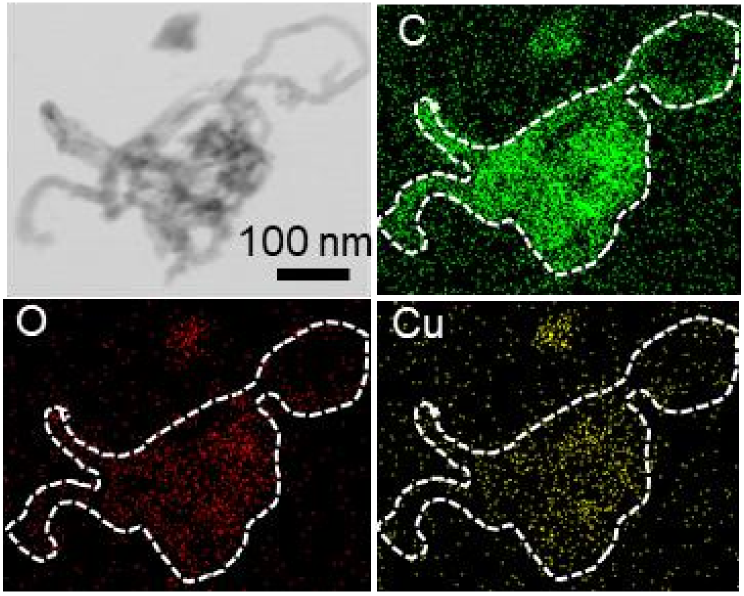

Figure S3. TEM element mapping of CNT-CuHHTP.

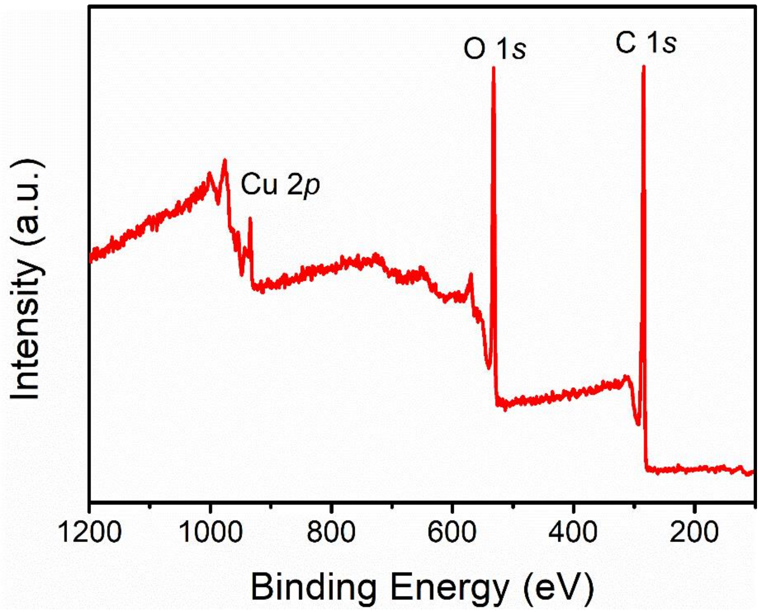

Figure S4 The XPS curves of CNT-CuHHTP.

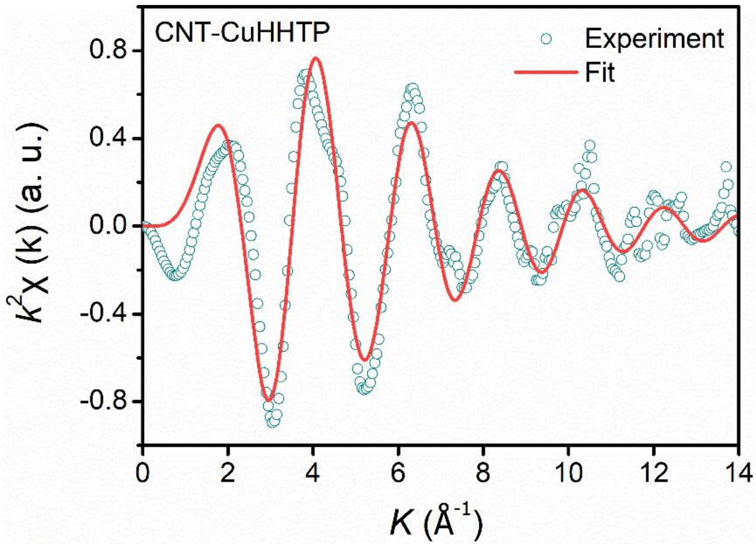

Figure S5 The Cu K-edge EXAFS fitting results for CNT-CuHHTP.

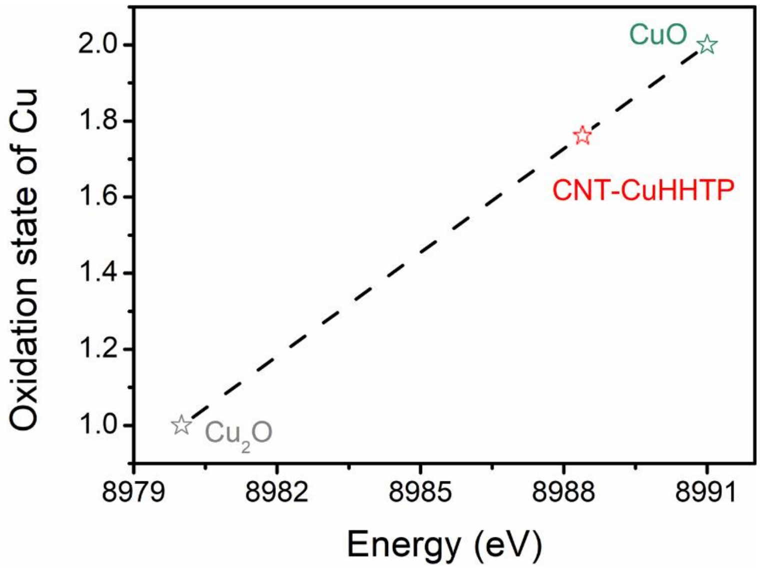

Figure S6 Oxidation state of Cu species for Cu_2O, CuO, and CNT-CuHHTP.

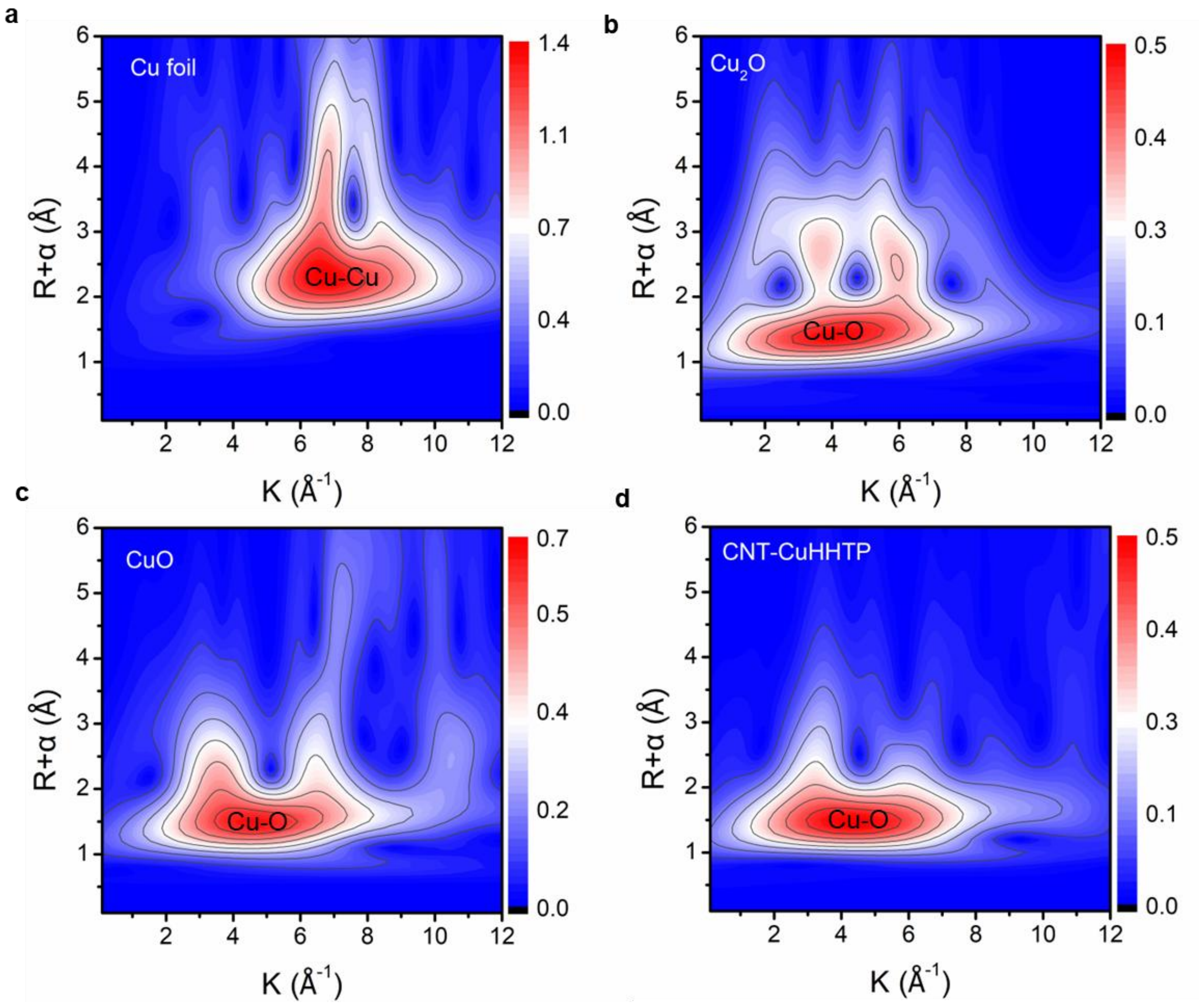

Figure S7 Wavelet transforms of Cu K-edge EXAFS data of Cu foil (**a**), Cu_2O (**b**), CuO (**c**) and CNT-CuHHTP (**d**).

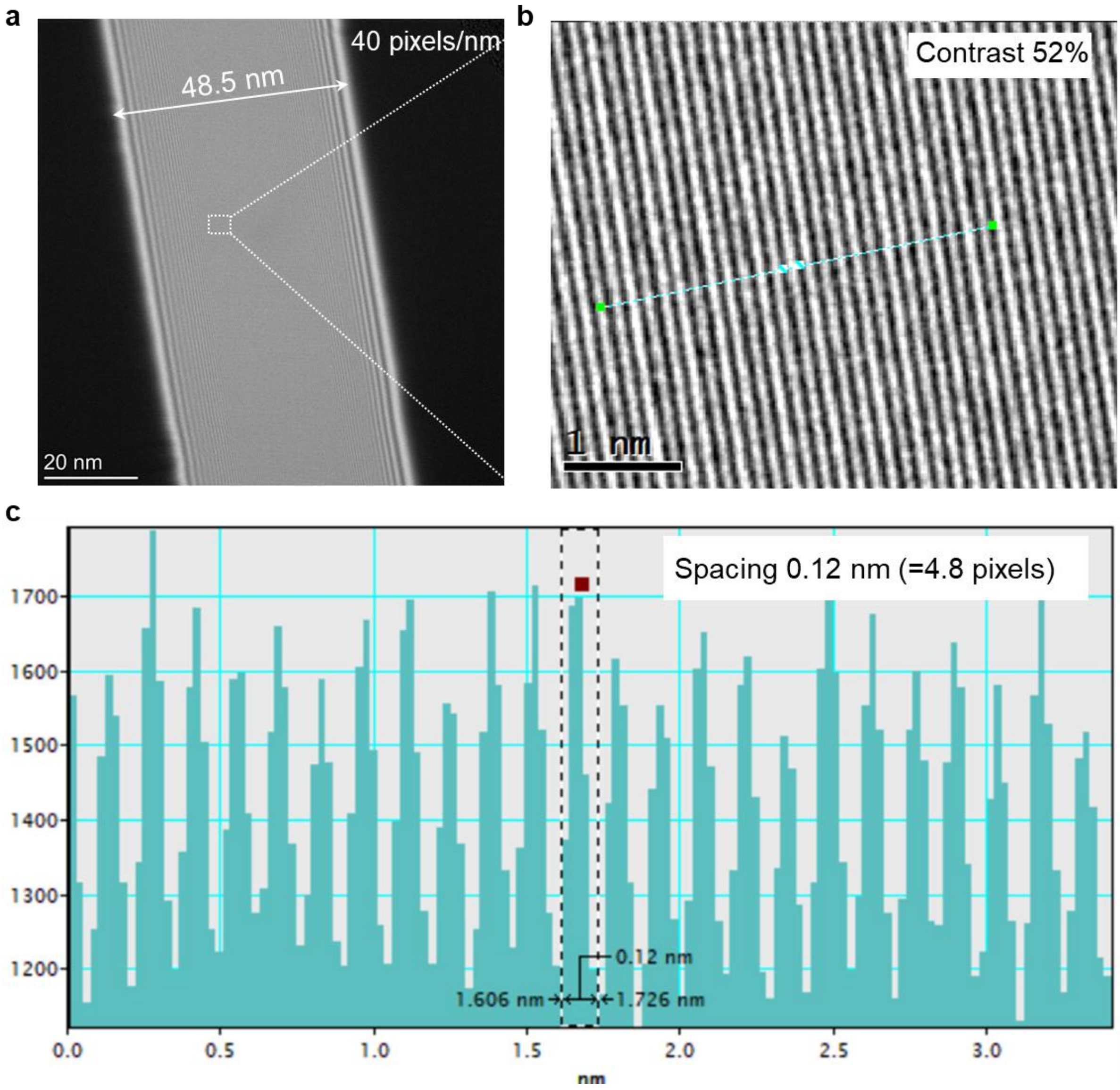

Figure S8 Hologram with electron beam traveling only through vacuum. **a,** Wide view hologram. **b,** Intensity profile obtained from region corresponding to **a**. **c,** Intensity profile obtained from region corresponding to **b**.

The spatial resolution

The field of view in Figure S 8a is 48.5 nm and the resolution 40 pixels/nm. The intensity contrast in the zoomed region Figure S 8b is 52%, indicating a high-quality recording[1]. In our work, each fringe was sampled by 4.8 pixels (Figure S 8c) of the detector conforming to the holography standard[2]. And, the fringe spacing is 0.12 nm, implying lateral resolution of the reconstructed phase, amplitude and electric field maps of ~ 0.36 nm.

The voltage resolution

The detection limit (σ_φ) is calculated using the following equation (1):

$$\sigma_\varphi \approx \frac{\sqrt{2}}{C\sqrt{Ne}} \tag{1}$$

σ_φ was evaluated using the region of the hologram indicated in image Figure S8b. Where C is the fringe contrast, and N_e is the number of detected electrons contributing to a give point in the reconstructed phase map.

C is 52% in Figure S8b. And, as shown in Figure S8c, the average photon count per pixel is ~1425. Thus:

$$\sigma_\varphi \approx \frac{\sqrt{2}}{0.52\sqrt{1425}} = 0.072$$

The voltage detection limit (V_{limit}) is calculated using the following equation (2):

$$V_{limit} = \frac{\sigma_\varphi}{C_E\, t} \tag{2}$$

Where C_E is the interaction constant (0.00728 rad V^{-1} nm^{-1} for 200 kV accelerating voltage), t is the thickness of sample. Because the fringe contrast decreases with thickness, the voltage detection limit is approximately independent of sample thickness[3]. Thus, the V_{limit} was ~ 0.1 V in our work.

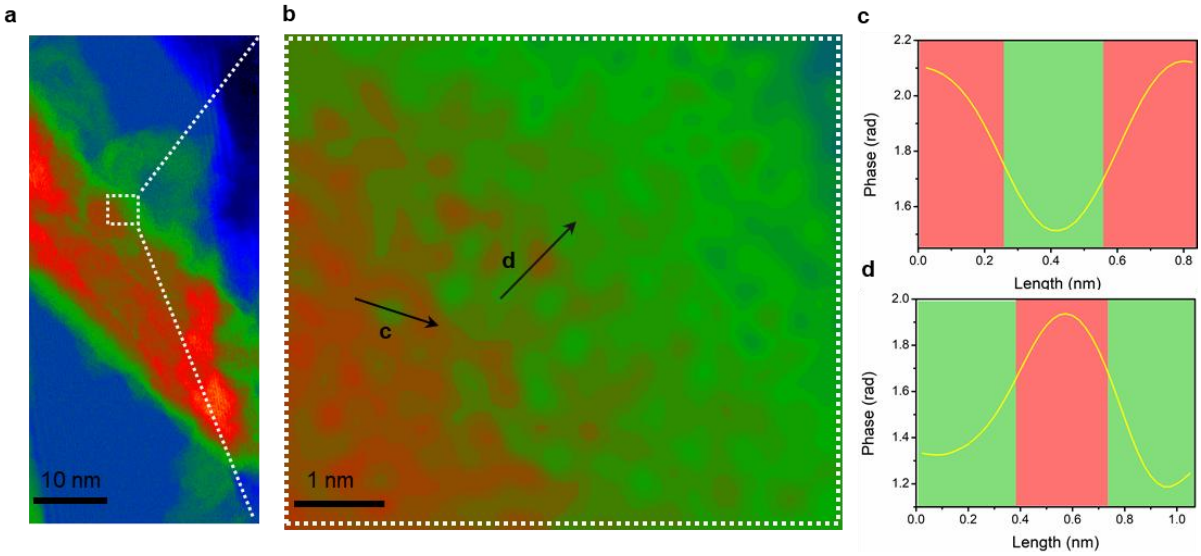

Figure S9 **a,** Reconstructed phase image of CNT-CuHHTP. **b,** Enlarged view of the white dashed box area in **a**. **c, d,** The profile of phase distribution corresponding to the black arrow region in **b**.

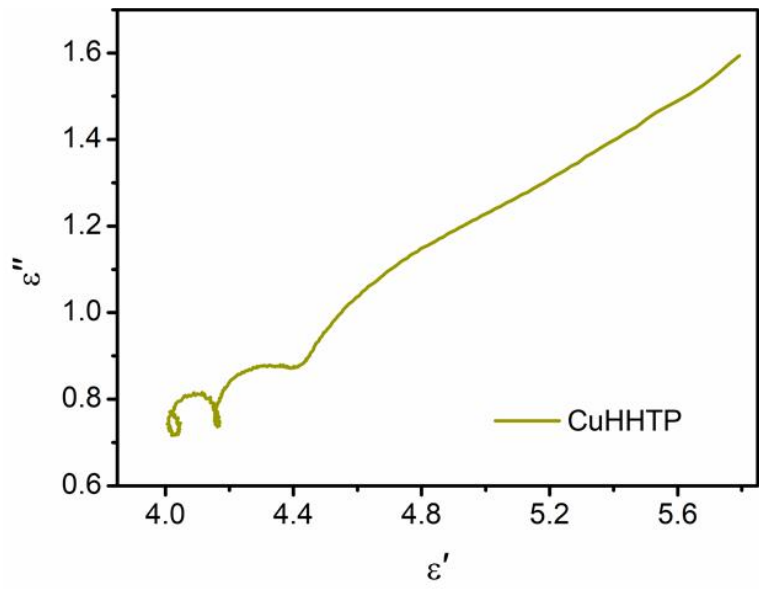

Figure S10 Plots of ε' versus ε'' for CuHHTP.

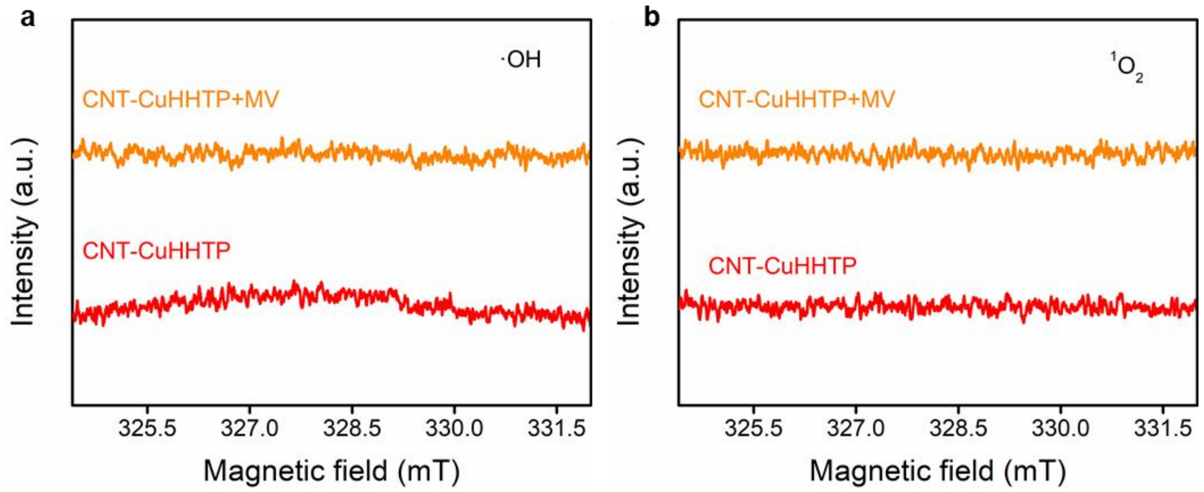

Figure S11. EPR measurements of ·OH (**a**) and $^1O_2$ (**b**) of CNT-CuHHTP before and after MV irradiation.

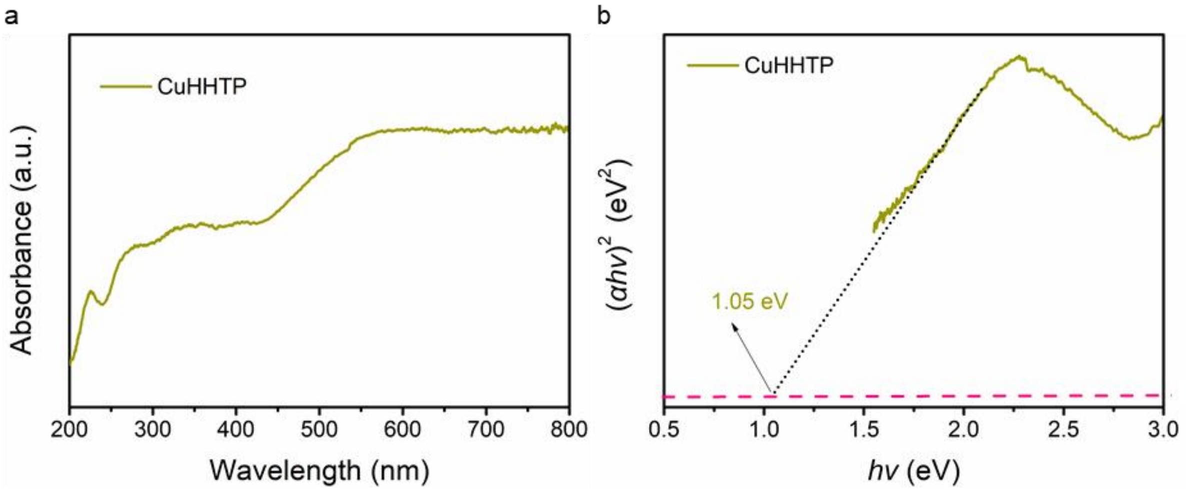

Figure S12 **a**, UV−vis diffuse reflectance spectra. **b**, Plots of $(\alpha h\nu)^2$ versus photon energy ($h\nu$).

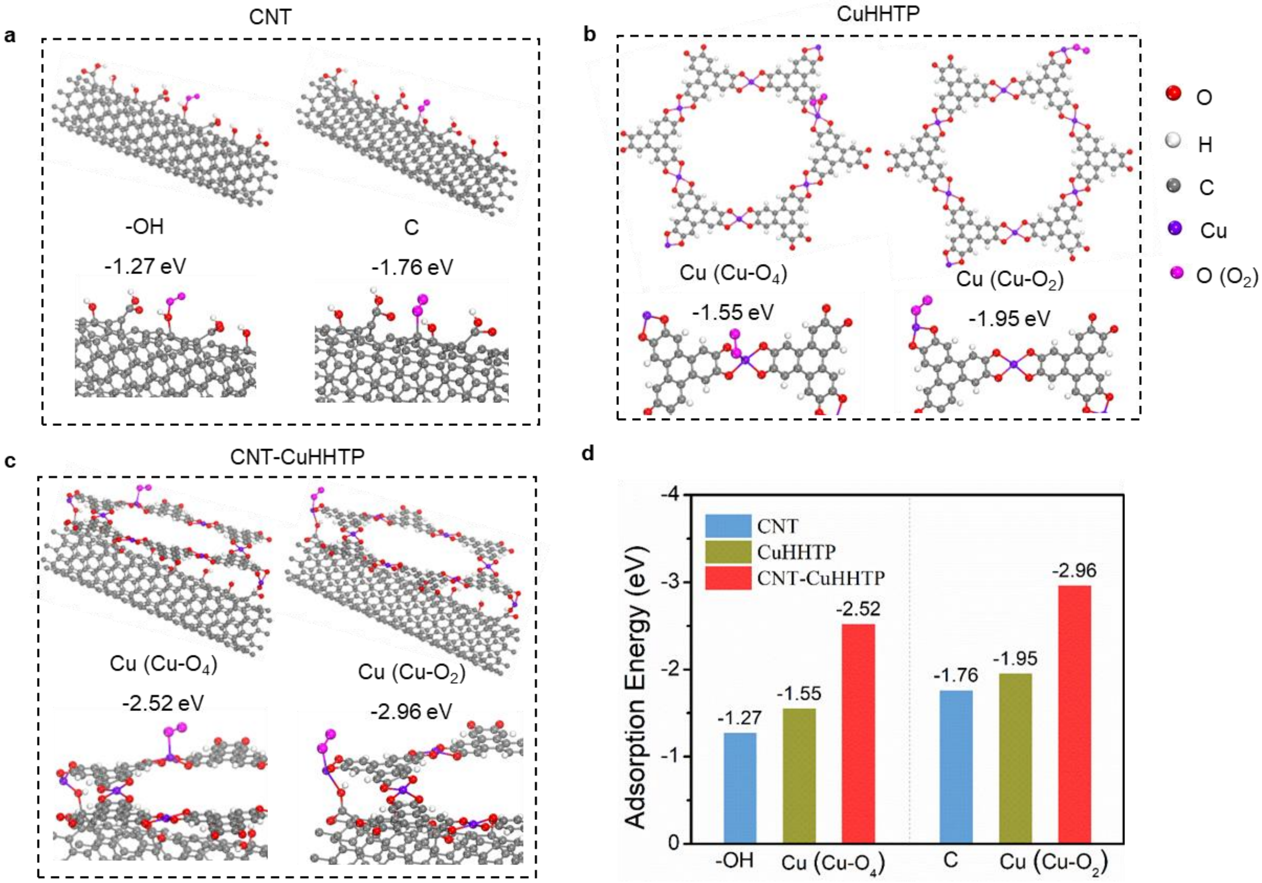

Figure S13 Adsorption sites and corresponding adsorption energies of samples for oxygen. **a-c**, Adsorption sites and corresponding adsorption energies of CNT (**a**), CuHHTP (**b**), and CNT-CuHHTP (**c**) for oxygen. **d**, Summary of adsorption energies of samples for different adsorption sites of oxygen.

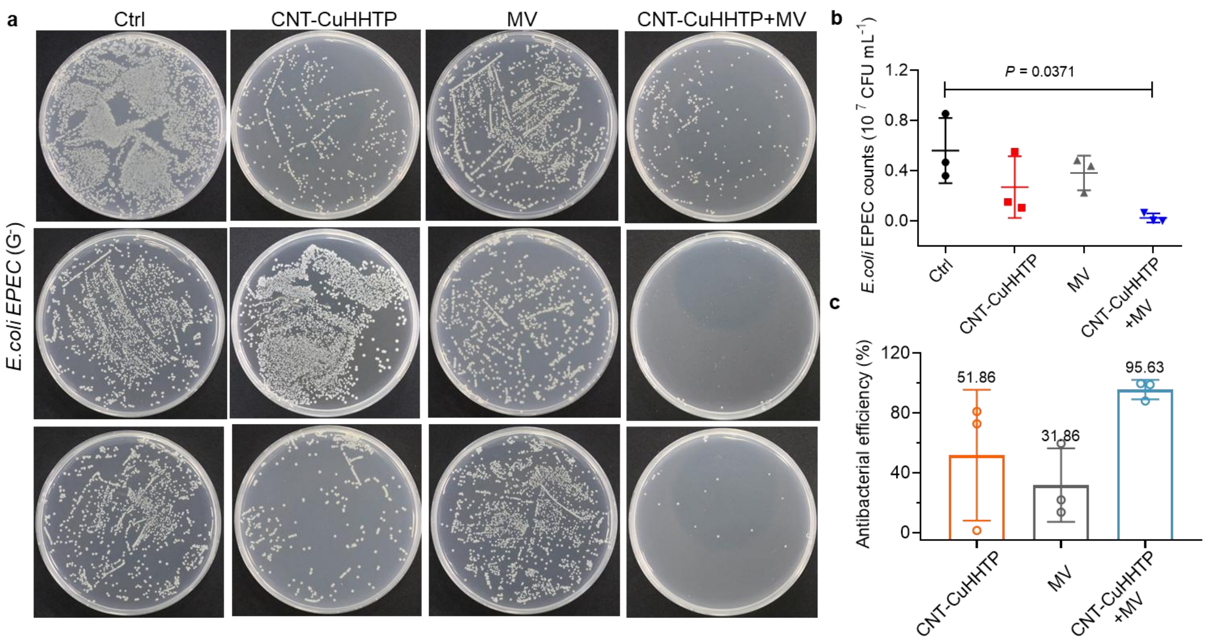

Figure S14 The efficacy of CNT-CuHHTP and MV synergistically killing *E. coli* EPEC. **a,** Spread-plate of *E. coli* EPEC treated with CNT-CuHHTP with/without 7 min MW irradiation (0.1 W cm^{-2}). **b,** *E. coli* EPEC strain counts calculated from spread-plate assays. **c,** Antibacterial efficiency of CNT-CuHHTP against *E. coli* EPEC with/without 7 min MW irradiation. Data are presented as mean ± standard deviations from a representative experiment ($n = 3$ independent samples). *P* values were analysed by one-way ANOVA with Tukey's multiple comparisons post hoc test. Significance was defined as $P \leq 0.05$.

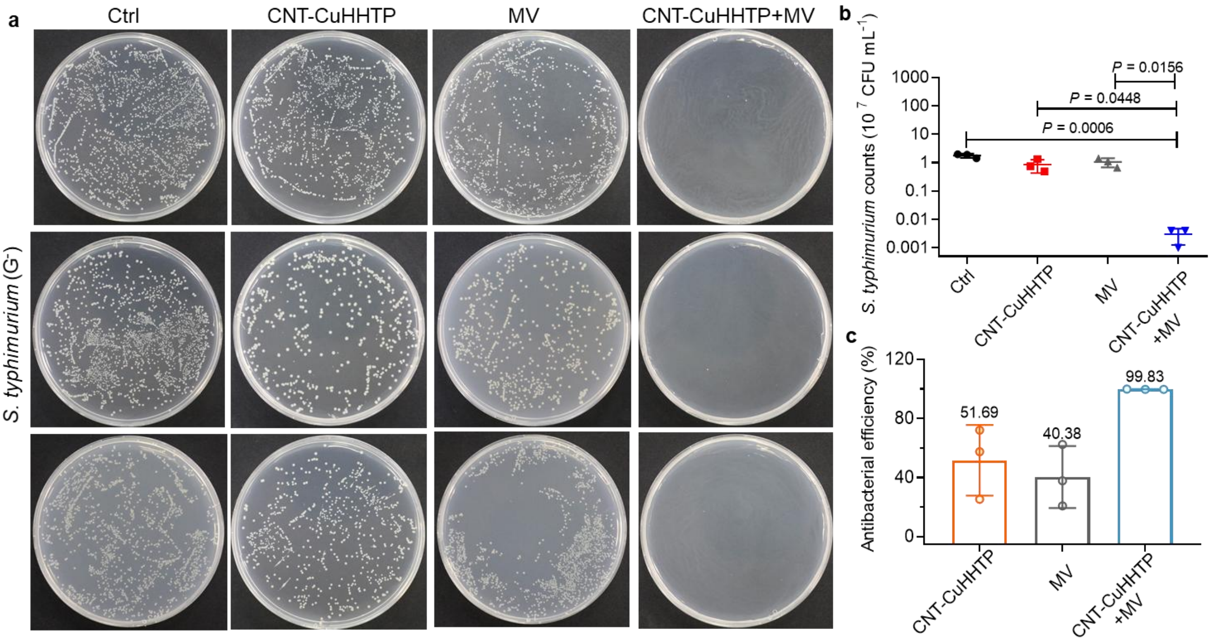

Figure S15 The efficacy of CNT-CuHHTP and MV synergistically killing *S. typhimurium*. **a,** Spread-plate of *S. typhimurium* treated with CNT-CuHHTP with/without 7 min MW irradiation (0.1 W cm^{-2}). **b,** *S. typhimurium* strain counts calculated from spread-plate assays. **c,** Antibacterial efficiency of CNT-CuHHTP against *S. typhimurium* with/without 7 min MW irradiation. Data are presented as mean ± standard deviations from a representative experiment ($n = 3$ independent samples). *P* values were analysed by one-way ANOVA with Tukey's multiple comparisons post hoc test. Significance was defined as $P \leq 0.05$.

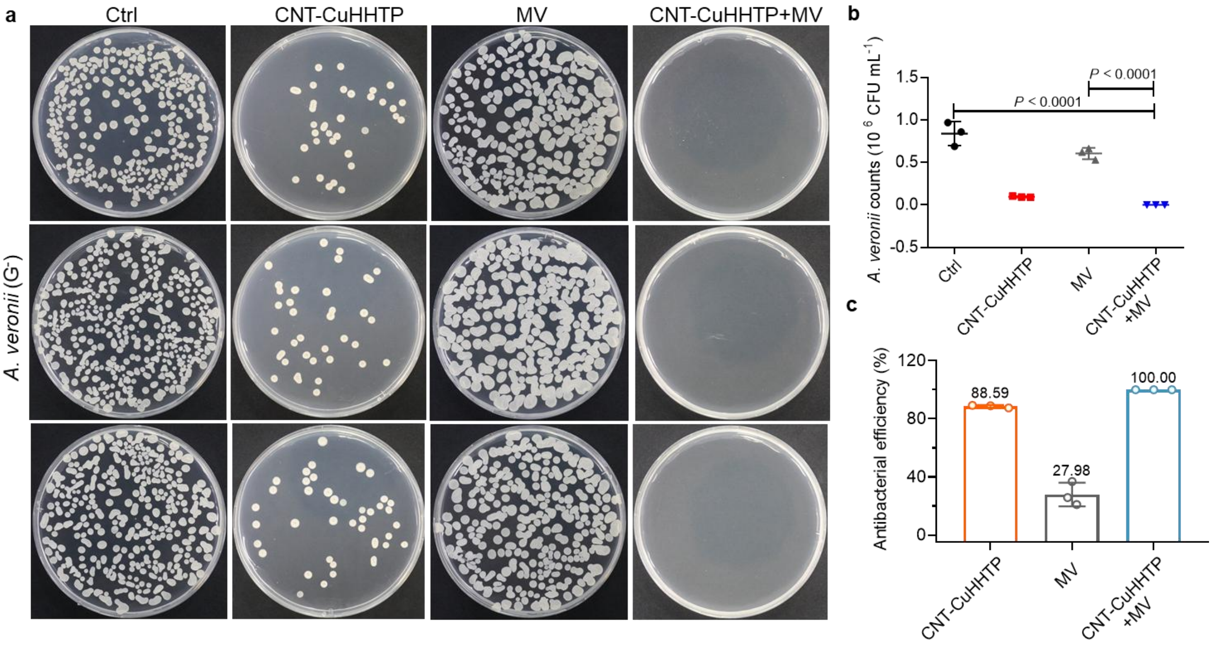

Figure S16 The efficacy of CNT-CuHHTP and MV synergistically killing *A. veronii*. **a,** Spread-plate of *A. veronii* treated with CNT-CuHHTP with/without 7 min MW irradiation (0.1 W cm^{-2}). **b,** *A. veronii* strain counts calculated from spread-plate assays. **c,** Antibacterial efficiency of CNT-CuHHTP against *A. veronii* with/without 7 min MW irradiation. Data are presented as mean ± standard deviations from a representative experiment ($n = 3$ independent samples). *P* values were analysed by one-way ANOVA with Tukey's multiple comparisons post hoc test. Significance was defined as $P \leq 0.05$.

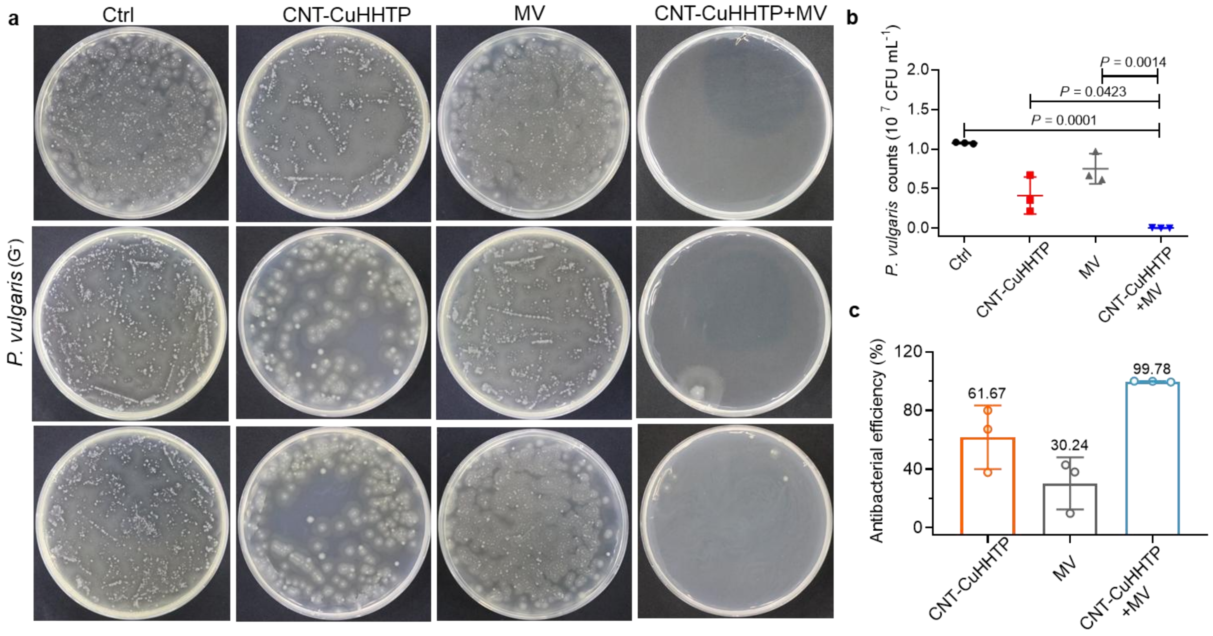

Figure S17 The efficacy of CNT-CuHHTP and MV synergistically killing *P. vulgaris*. **a,** Spread-plate of *P. vulgaris* treated with CNT-CuHHTP with/without 7 min MW irradiation (0.1 W cm^{-2}). **b,** *P. vulgaris* strain counts calculated from spread-plate assays. **c,** Antibacterial efficiency of CNT-CuHHTP against *P. vulgaris* with/without 7 min MW irradiation. Data are presented as mean ± standard deviations from a representative experiment ($n = 3$ independent samples). P values were analysed by one-way ANOVA with Tukey's multiple comparisons post hoc test. Significance was defined as $P \leq 0.05$.

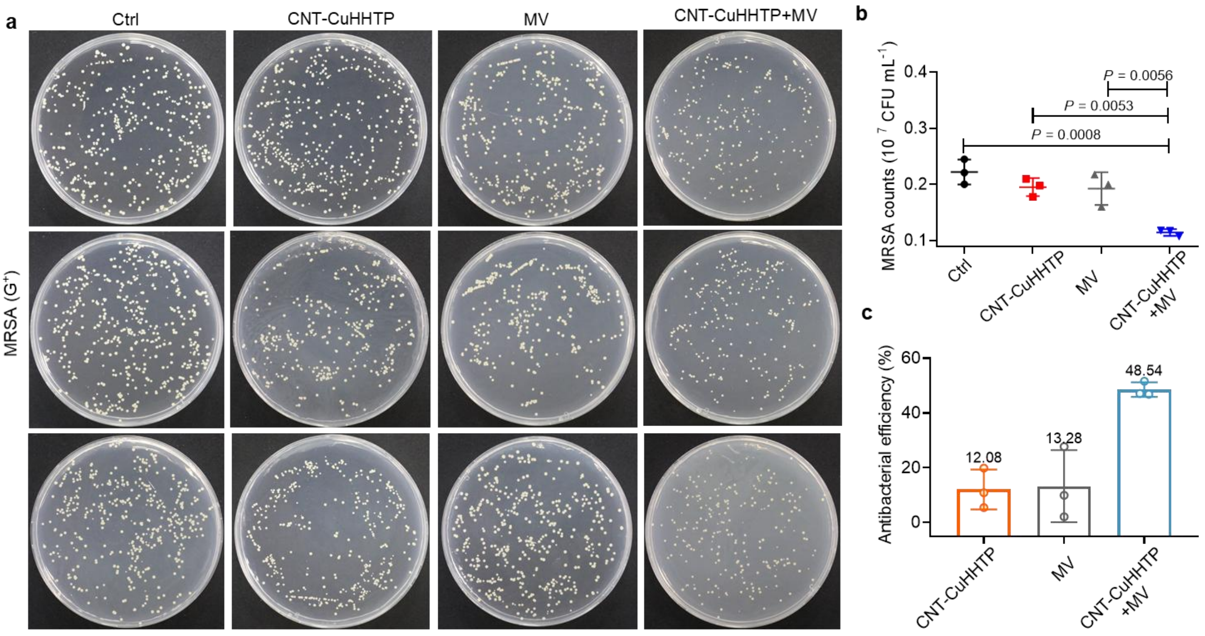

Figure S18 The efficacy of CNT-CuHHTP and MV synergistically killing MRSA. **a,** Spread-plate of MRSA treated with CNT-CuHHTP with/without 7 min MW irradiation (0.1 W cm^{-2}). **b,** MRSA strain counts calculated from spread-plate assays. **c,** Antibacterial efficiency of CNT-CuHHTP against MRSA with/without 7 min MW irradiation. Data are presented as mean ± standard deviations from a representative experiment ($n = 3$ independent samples). P values were analysed by one-way ANOVA with Tukey's multiple comparisons post hoc test. Significance was defined as $P \leq 0.05$.

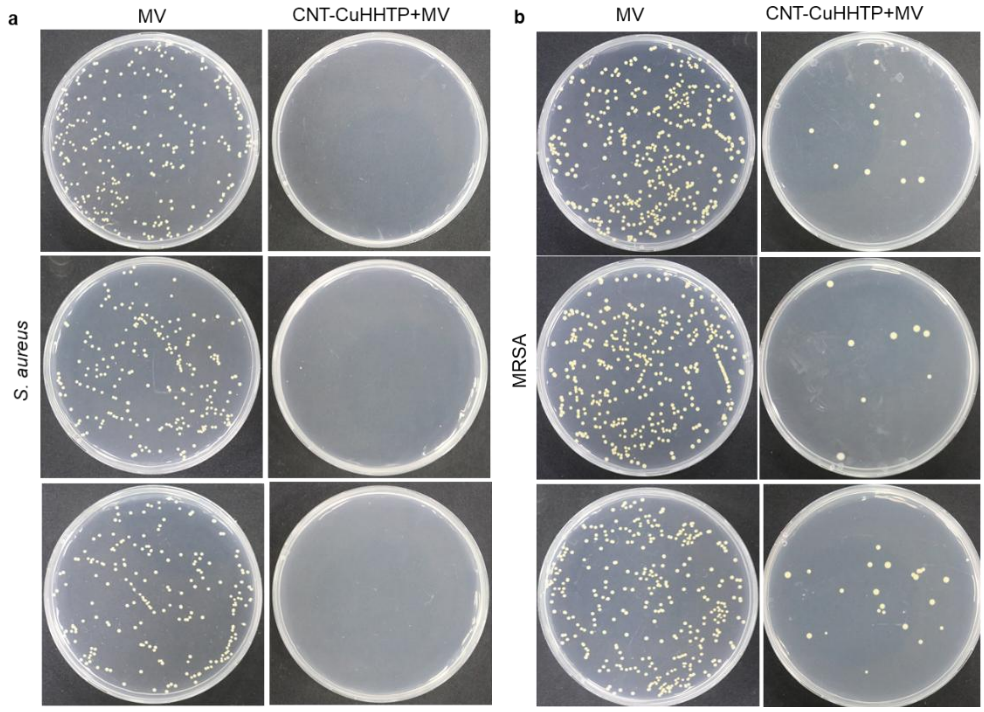

Figure S19 Antibacterial effect of CNT-CuHHTP on *S. aureus* and MRSA after increasing MV power. **a,** Spread-plate of *S. aureus* treated with 7 min MW irradiation (0.13 W cm^{-2}) with/without CNT-CuHHTP. **b,** Spread-plate of MRSA treated with7 min MW irradiation (0.13 W cm^{-2}) with/without CNT-CuHHTP.

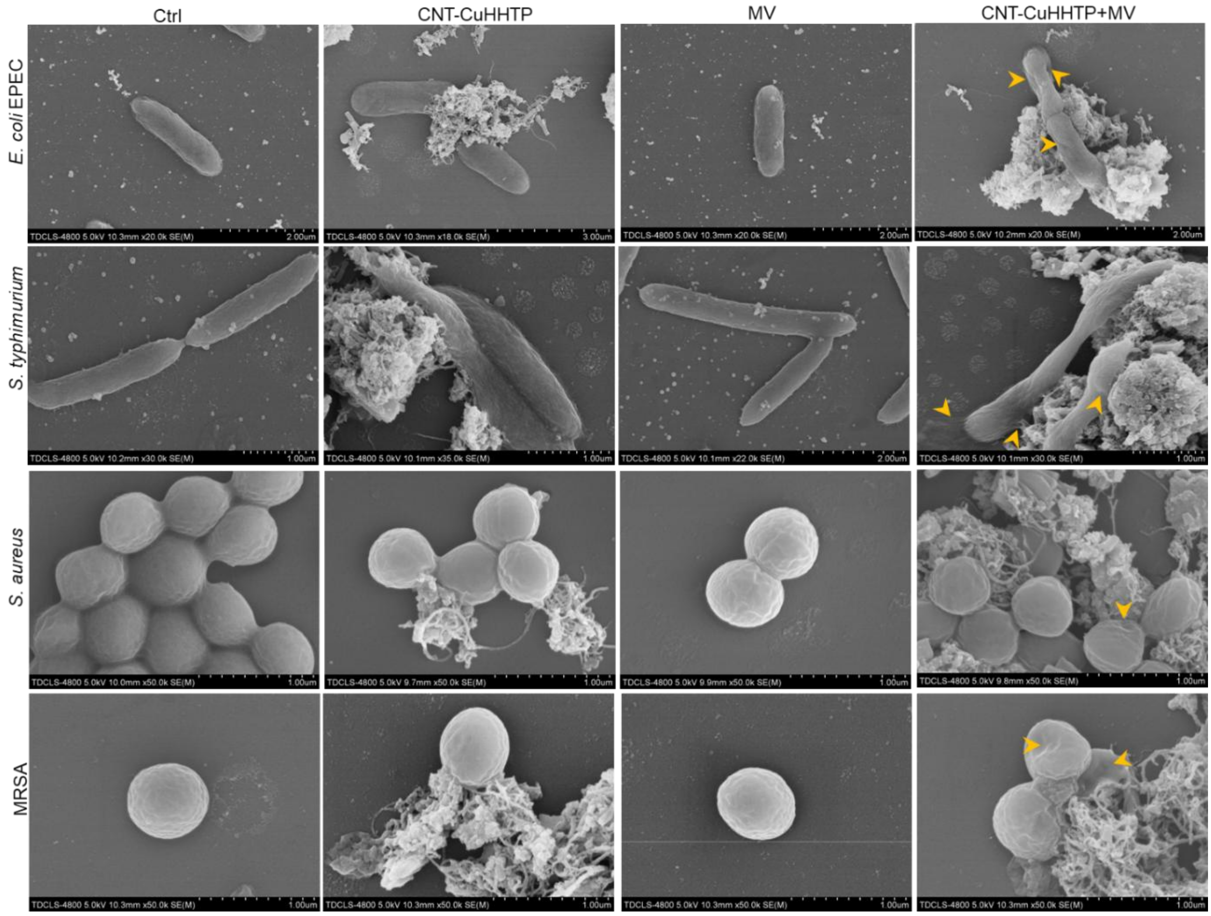

Figure S20 SEM images of *E. coli* EPEC, *S. typhimurium*, *S. aureus*, and MRSA treated with different conditions.

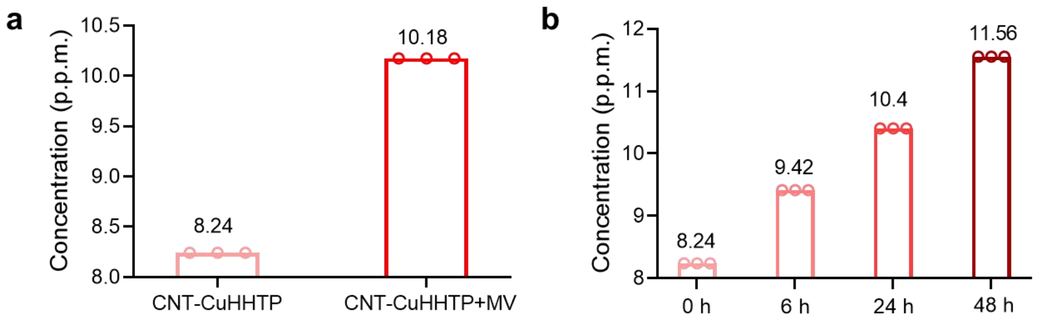

Figure S21 **a,** Copper ion release from CNT-CuHHTP before and after microwave irradiation. **b,** The release of copper ions from CNT-CuHHTP within 48 hours. The concentration of CNT-CuHHTP is 1 mg mL^{-1} consistent with the antibacterial test conditions. n = 3 repeated tests.

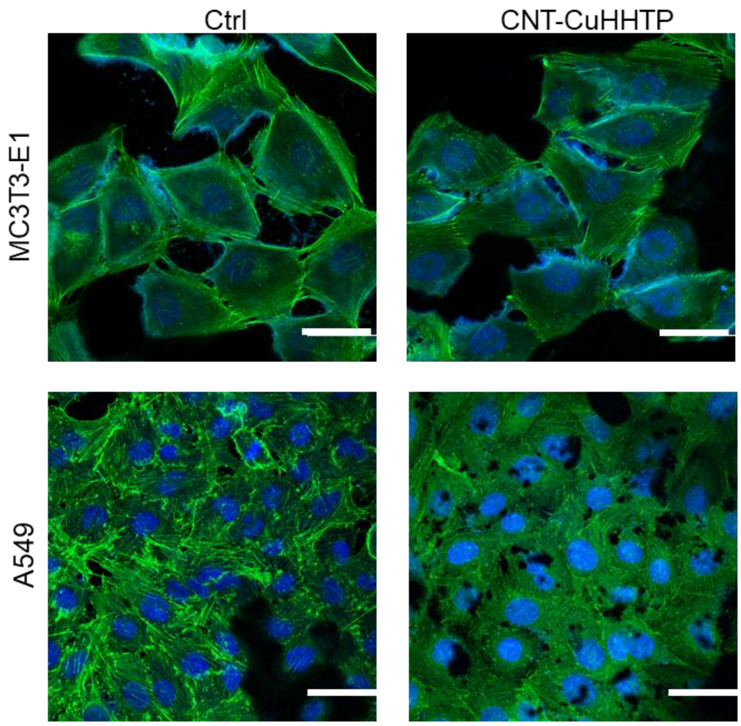

Figure S22 Fluorescent images of MC3T3-E1 osteoblasts or A549 cocultured with or without CNT-CuHHTP for three days, respectively. Scale bar, 50 μm.

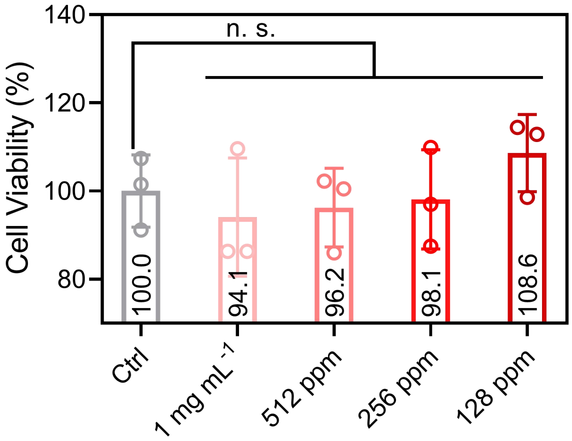

Figure S23 The viability of MC3T3-E1 cells cocultured with different concentrations of CNT-CuHHTP after coculturing for three days. $n = 3$ independent samples. The n.s. present $P > 0.05$, and P values were analysed by one-way ANOVA with Dunnett's multiple comparisons post hoc test.

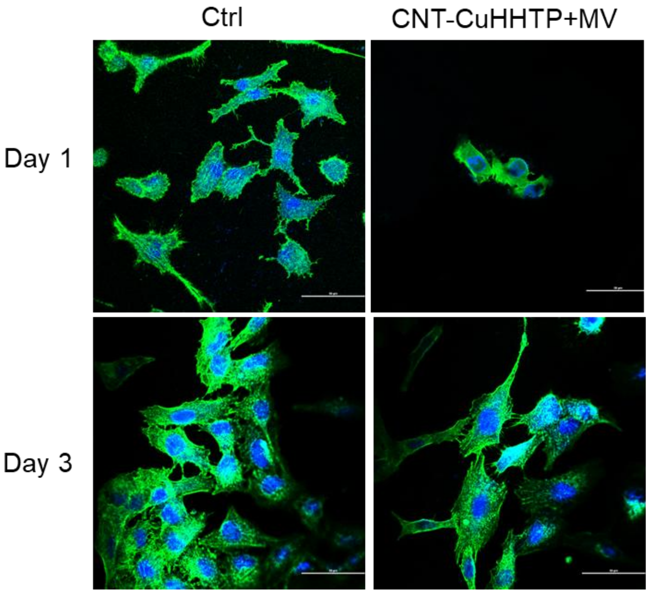

Figure S24 Fluorescent images of A549 were cultured for one and three days after treatment of CNT-CuHHTP+MV or not. Scale bar, 50 μm.

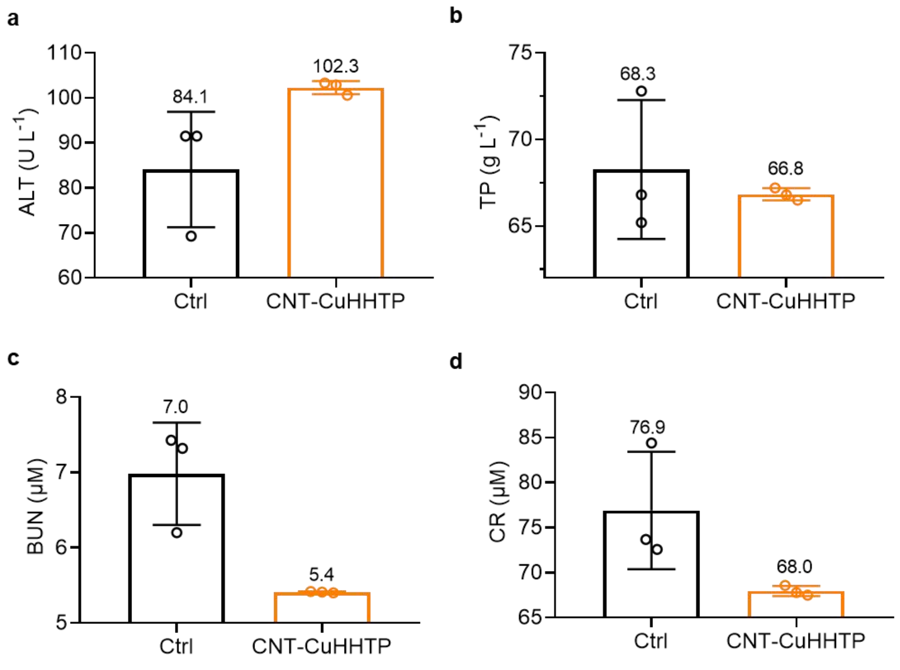

Figure S25 **a, b,** Hepatic function (ALT and TP) of Ctrl and CNT-CuHHTP groups on day 5. **c, d,** Renal function (BUN, CR, and UA) of Ctrl and CNT-CuHHTP groups on day 5. $n = 3$ independent samples.

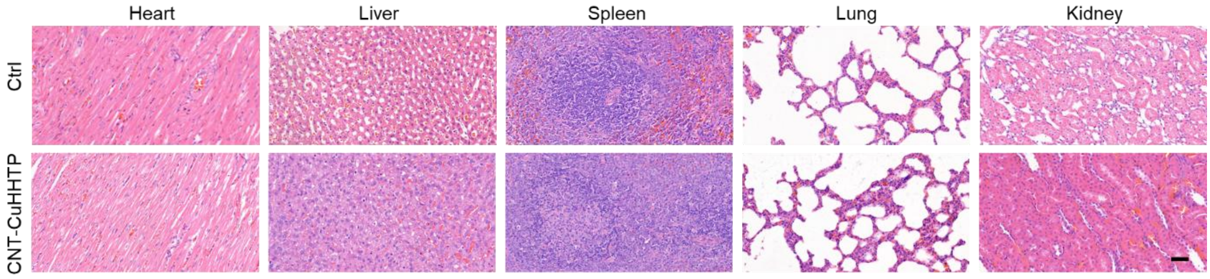

Figure S26 HE sections of major organs. Scale bar, 50 μm.

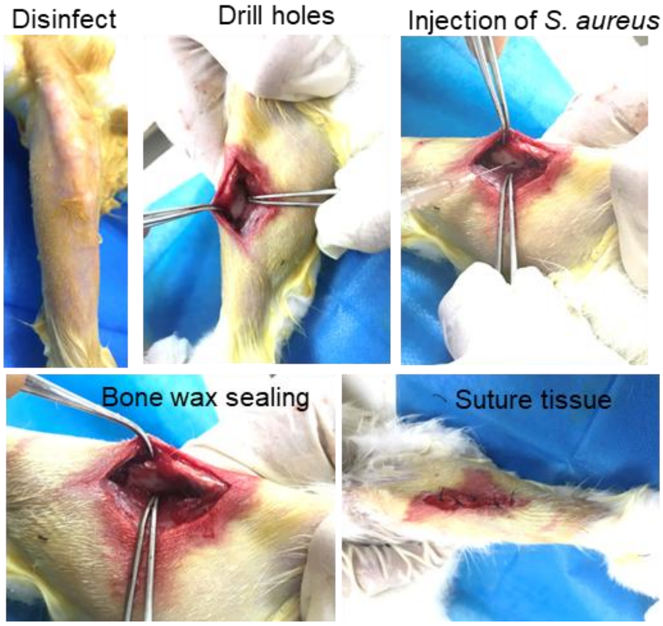

Figure S27 Surgical procedure of rabbit osteomyelitis model.

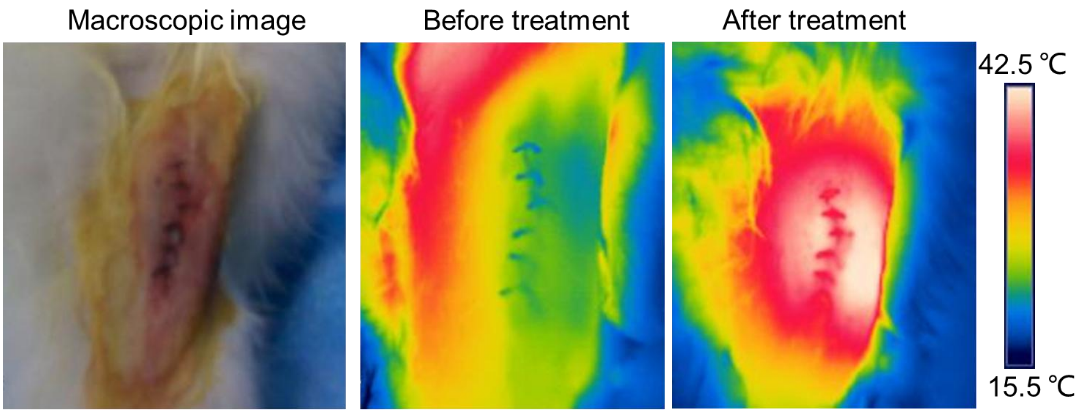

Figure S28 Thermal imaging pictures of rabbit bone tissue before and after CNT-CuHHTP+MV treatment.

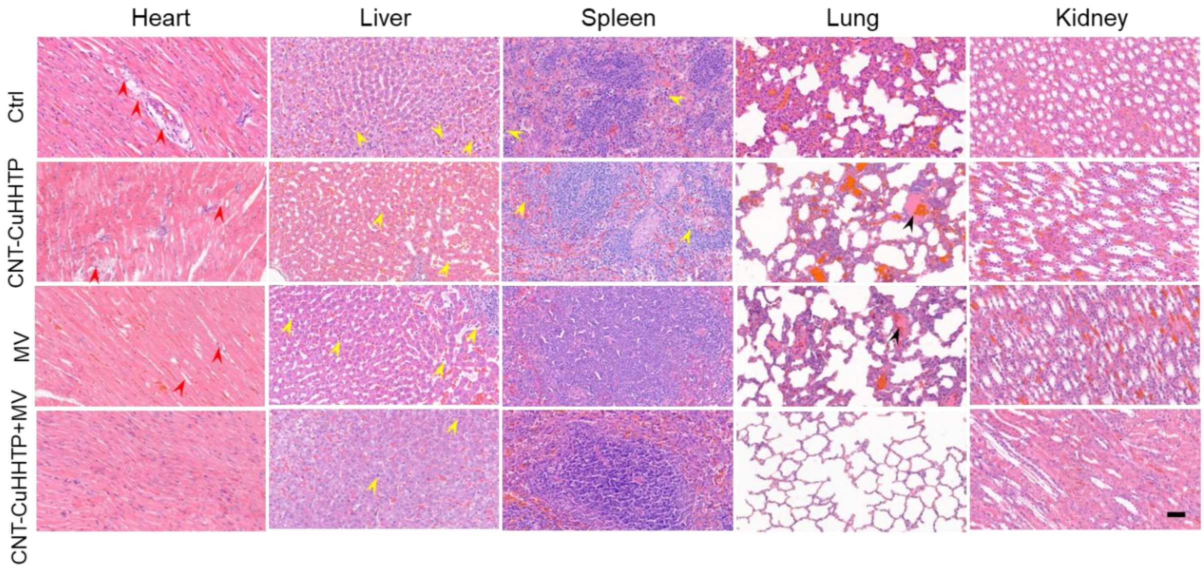

Figure S29 H&E staining images of heart, liver, spleen, lung, and kidney after 5 days posttreatment. Scale bar, 50 μm.

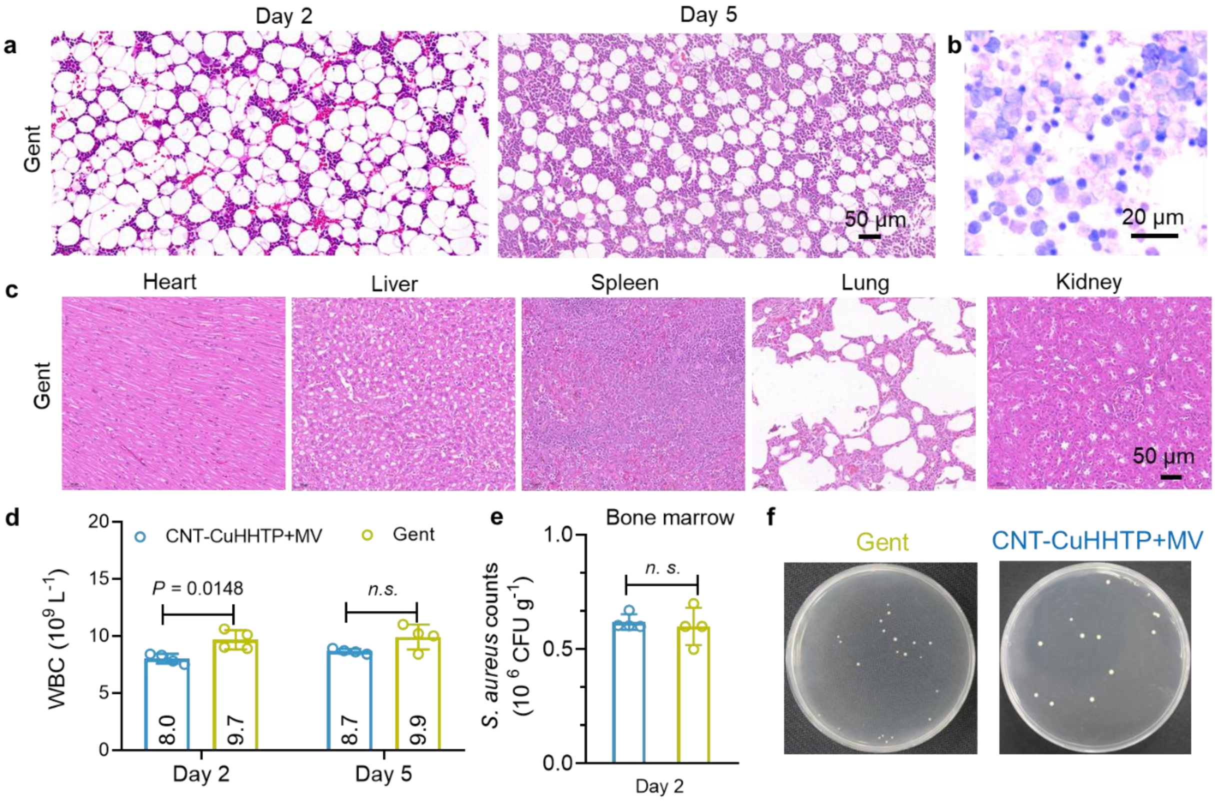

Figure S30 **a,b,** H&E staining (**a**) and Wright-stained (**b**) images of bone marrow in Gent group. **c,** H&E staining images of heart, liver, spleen, lung, and kidney after 5 days Gent treatment. **d,** Amount of WBC at 2 and 5 days in blood. Data are presented as mean ± standard deviations from a representative experiment ($n = 4$ independent samples). P values were analysed by two-way ANOVA with Sidak's multiple comparisons post hoc test. **e**, The *S. aureus* counts in the bone marrow after 2 days with different treatments. Data are presented as mean ± standard deviations from a representative experiment ($n = 4$ independent samples). P values were analysed by t test. **f,** Spread-plate of *S. aureus* in the bone marrow after treated with CNT-CuHHTP+MW or Gent. Significance was defined as $P \leq 0.05$. The *n.s.* present no significant difference.

We selected gentamicin (Gent), which is commonly used in clinical treatment of osteomyelitis, as the antibiotic treatment group (positive control) to compare the effectiveness between the CNT-CuHHTP+MW therapy and traditional antibiotic therapy. After Gent treatment, HE staining (Figure S30 a) of rabbit bone marrow showed that its cell morphology was normal, inflammatory cells were few, and the degree of adipocyte damage was very low, which was consistent with the results of Wright staining (Figure S30 b). In addition, the main organs of the rabbit were not infected or damaged (Figure S30 c). These results fully prove the effectiveness of Gent in the treatment of osteomyelitis.

Besides, after 2 days of surgery, the WBC (*P* = 0.0148) levels of the CNT-CuHHTP+MW group were significantly lower than that the Gent group, the bacterial infection was restrained in the CNT-CuHHTP+MW group due to the synergistic treatment of CNT-CuHHTP+MW. And, there is no statistical difference in WBC level between the group of CNT-CuHHTP+MW and Gent after 5 days of surgery (Figure S30 d). This indicates that the therapeutic effect of CNT-CuHHTP+MW is similar to that of Gent. To quantify the antibacterial properties of CNT-CuHHTP+MW, we performed the colony-count assay using the harvested bone marrow tissues (Figure S30 e, f). The number of *S. aureus in* the group of CNT-CuHHTP+MW is as low as that in group of Gent. These results proved that the improvement effect of CNT-CuHHTP+MV on osteomyelitis is comparable to that of Gent.

Table S1. EXAFS fitting parameters at the Cu *K*-edge for various samples (S_0^2=0.71).

Sample	Shell	*CN*	*R* (Å)	σ^2 (Å^2)	ΔE_0 (eV)	R factor (%)
Cu *K*-edge						
Cu_2O	Cu-O	2*	1.86±0.016	0.0019	7.34±1.24	0.6
CuO	Cu-O	4*	1.94±0.011	0.0053	3.03±0.27	0.8
	Cu-Cu	4*	2.92±0.024	0.0083	5.30±2.10	
CNT-CuHHTP	Cu-O	3.46±0.52	1.94±0.03	0.0044	3.38±2.19	1.4

CN, coordination number; *R*, distance between absorber and backscatter atoms; σ^2, Debye-Waller factor to account for both thermal and structural disorders; ΔE_0, inner potential correction; *R* factor indicates the goodness of the fit. S_0^2 was fixed to 0.71, according to the experimental EXAFS fit of Cu foil by fixing CN as the known crystallographic value. Fitting range: $3.0 \leq k$ (Å^{-1}) ≤ 12 and $1.5 \leq R$ (Å) ≤ 3.0 (Cu foil); $3.0 \leq k$ (Å^{-1}) ≤ 11.0 and $1.0 \leq R$ (Å) $\leq$ ~2.2 (CNT-CuHHTP). A reasonable range of EXAFS fitting parameters: $0.700 < S_0^2 < 1.000$; $CN > 0$; $\sigma^2 > 0$ Å^2; $\Delta E_0 < 10$ eV; *R* factor < 0.02.

1. Niermann T, Lehmann M. Holographic focal series: differences between inline and off-axis electron holography at atomic resolution. *Journal of Physics D: Applied Physics* **2016**, 49, 194002.

2. Lichte H. Performance limits of electron holography. *Ultramicroscopy* **2008**, 108, 256-262.

3. Xu X*, et al.* Variability and origins of grain boundary electric potential detected by electron holography and atom-probe tomography. *Nature Materials* **2020**, 19, 887-893.
